# Evolution of enlarged body size of coal tits *Parus ater* in geographic isolation from two larger competitors, the crested tit *Parus cristatus* and the willow tit *Parus montanus*, on six Scandinavian islands

**DOI:** 10.1242/bio.013839

**Published:** 2015-10-21

**Authors:** R. Åke Norberg, Ulla M. Lindhe Norberg

**Affiliations:** Department of Biological and Environmental Sciences – Zoology, University of Gothenburg, Gothenburg 40530, Sweden

**Keywords:** Parus ater, Character release, Coal tit, Competition, Evolution, Evolution on islands

## Abstract

Here, we report that on six widely separated Scandinavian islands, the coal tit *Parus ater* has evolved morphologically in the direction of two absent competitors, the crested tit *P. cristatus* and the willow tit *P. montanus*, to the effect that it is up to 10% larger in linear dimensions than conspecifics on the adjacent Swedish mainland, where all three species coexist. The large size is genetically determined, as ascertained by clutch exchange experiments between island and mainland nests. We conclude that the increased size of *P. ater* in places where it is geographically isolated from its larger congeners is the result of evolutionary adaptation, due ultimately to relaxed interspecific competition. On the islands, *P. ater* has evolved into a medium-sized generalist, with selection pressures likely governed by the following causal relationships. When competitors are lacking, *P. ater* takes over the foraging space of the absentees. The enlarged food base allows higher population densities, which intensifies intraspecific interference competition. This, in turn, selects for increased body size. When *P. ater* coexists with its larger congeners, it occupies peripheral foraging sites in trees, which requires excellent manoeuvrability and energy-expensive locomotion modes. Reduction of body size increases locomotor capacity for mechanical and aerodynamic reasons and lowers energy consumption, so small size is favoured in sympatry. But in geographic isolation, *P. ater* exploits the tree periphery less and the inner tree regions more, and it also adopts the easier locomotion modes of the absent species. Therefore, selection for manoeuvrability and a small body size is relaxed. The new selection regime shifts the balance between opposing selection forces towards a larger body size. We were able to test 11 alternative hypotheses and available evidence conclusively eliminates them all. As a result, here, evolution could be predicted regarding both direction and amount of change.

## Abstract (precis)

**Summary:** On six widely separated Scandinavian islands, an enlarged food base and intraspecific competition has caused the coal tit *Parus ater* to evolve morphologically in the direction of two absent competitors.

## INTRODUCTION

Adaptation to local conditions is a key process in the evolution of biological diversity. Here we explore whether reduced interspecific competition in island populations of the coal tit *Parus ater* has brought about any evolutionary change in ecologically important aspects of morphology.

**Fig. 1.**
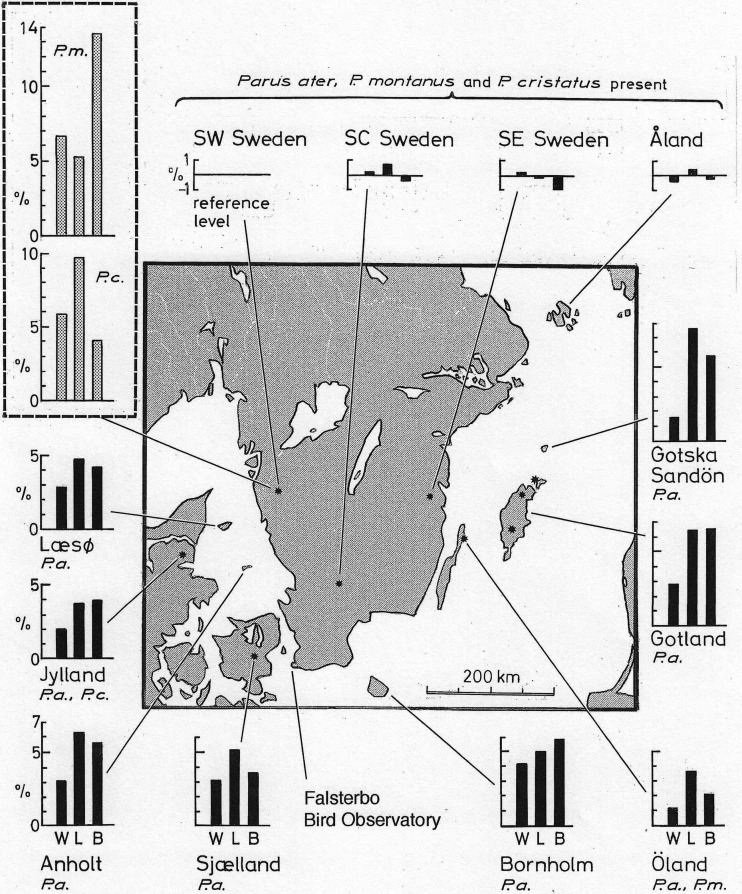
**Map of southern Sweden and adjacent islands showing the location of 12 sampling places and Falsterbo Bird Observatory.** The map reaches north to latitude 61°N, whereas the coniferous forest tits *P. ater* (*P.a.*), *P. cristatus* (*P.c.*) and *P. montanus* (*P.m.*) coexist on the Swedish mainland up to about 63°N. Measurements of *P. ater* in SW Sweden are used as reference values. Black bars show by how many percent *P. ater* at each of the 11 other places differs from *P. ater* in SW Sweden. Each bar represents an average difference as calculated across three measurements. The left bar (W) shows the average of the percentage difference from the SW Swedish birds in (wing area)^0.5^, wing span and hand-wing length. The middle bar (L) shows the average percentage difference across tibiotarsus, tarsus ‘short’ and foot. And the right bar (B) shows the average percentage difference across bill length, width and height. As indicated at the graphs, *P. ater* coexists with *P. montanus* and *P. cristatus* on the Swedish mainland and the Finnish island of Åland. And *P. ater* is the same size in these places. The larger competitors *P. cristatus* and *P. montanus* are absent from the islands Gotska Sandön, Gotland, Bornholm, Sjælland, Anholt and Læsø. And an evolutionary increase of the body size of *P. ater* has taken place on all six islands. *P. ater* coexists with *P. cristatus* on the Danish penisnsula Jylland and with *P. montanus* on the island of Öland*.* The size of *P. ater* has increased also in these places, but not as much as where both *P. cristatus* and *P. montanus* are absent. The inset at top left shows how much *P. montanus* and *P. cristatus* from SW and SE Sweden differs from the reference values for *P. ater* in SW Sweden.

We follow the nomenclature of Dickinson (2003) with regard to the genus *Parus*. The mainland of Sweden and Norway south of latitude 61°N, covered by the map in Fig. 1, is inhabited by six *Parus* species. Three of them, the coal tit *P. ater*, the crested tit *P. cristatus* and the willow tit *P. montanus* are strictly confined to coniferous forest, which is their preferred habitat, except that *P. montanus* also inhabits the alpine birch zone (*Betula spp.*) in northernmost Scandinavia (Haftorn, 1954, 1956a,b,c; Ekman, 1979; SOF, 1990). The other three, the great tit *Parus major*, the marsh tit *P. palustris* and the blue tit *P. caeruleus* are bound to deciduous forests*.* Because of their different habitat requirements there is little interference between the two sets of species. The habitat preferences described above refer to Scandinavia.

The three coniferous forest tit species regularly occur together in mixed-species foraging flocks outside the breeding season (Haftorn, 1954, 1956a,b,c; Ekman, 1979). In winter, this foraging guild often contains also the treecreeper *Certhia familiaris* and the goldcrest *Regulus regulus*. These five species are separated ecologically mainly by their different foraging zones in trees (Alatalo, 1980, 1981, 1982a; Alatalo et al., 1985; Alerstam et al., 1974; Haftorn, 1956c; Hogstad, 1978; Lack, 1971). Within the respective foraging zone, each species uses its own set of locomotion modes and feeding postures. The three *Parus* species have fairly similar diet though, both regarding food items stored in autumn for consumption in winter and food given to nestlings (Haftorn, 1954; Haftorn, 1956a,b,c; von Brömssen and Jansson, 1975). There is overwhelming evidence for interspecific competition over foraging sites and food between the three coniferous forest tits in winter (Askenmo et al., 1977; Ekman et al., 1981; Jansson et al., 1981; Alatalo, 1982b).

On the mainland of Norway, Sweden and Finland, *P. ater*, *P. cristatus* and *P. montanus* regularly occur together in coniferous forest regions south of latitude 62°–63°N. But on six major islands off the Swedish coast the two largest species *P. cristatus* and *P. montanus* are lacking, so *P. ater* is the only coniferous forest tit (Table 1). This offers exceptionally good opportunities for testing whether relaxed interspecific competition on the islands has had any evolutionary effect on the eco-morphology of *P. ater*.

**Table 1.**
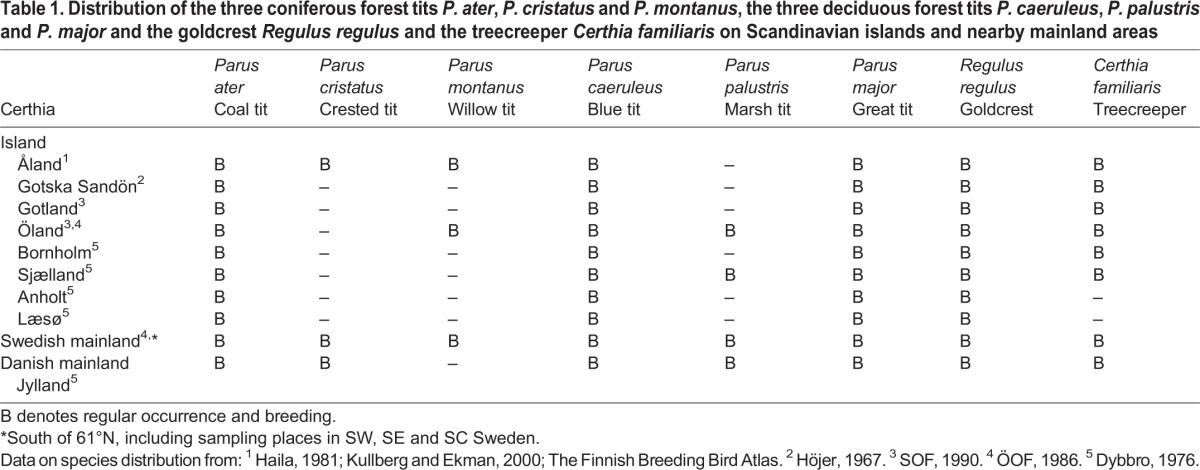
**Distribution of the three coniferous forest tits *P. ater*, *P. cristatus* and *P. montanus*, the three deciduous forest tits *P. caeruleus*, *P. palustris* and *P. major* and the goldcrest *Regulus regulus* and the treecreeper *Certhia familiaris* on Scandinavian islands and nearby mainland areas**

Ecologically important aspects of morphology, and in particular the overall body size, are co-adapted with the characteristic feeding sites, body postures and locomotion modes used by the respective tit species during foraging (Palmgren, 1932; Haftorn, 1956c; Lack, 1971; Norberg, 1979; Moreno and Carrascal, 1993). When *P. ater* coexists with its larger congeners, it occupies peripheral foraging sites in trees, which requires excellent manoeuvrability and energy-expensive locomotion modes. For mechanical and aerodynamic reasons, small birds have better locomotor capacity and lower energy costs for locomotion than larger ones (Andersson and Norberg, 1981), so small size is favoured in sympatry.

On the island of Gotland, where *P. cristatus* and *P. montanus* are naturally absent, *P. ater* has extended its foraging niche and exploits also the inner tree zones that are normally used by the absentees (Alerstam et al., 1974; Gustafsson, 1988). Partial niche takeover occurred also in experimental forest plots on the mainland of east-central Sweden; after the population density of *P. cristatus* and *P. montanus* had been experimentally reduced by more than 50%, their foraging zones in the inner canopy parts were used more frequently by *P. ater* and *R. regulus* (Alatalo et al., 1985). And an aviary experiment, which tested *P. ater* and *P. montanus* against each other, showed that when *P. montanus* tits were removed, *P. ater* turned to exploiting also the characteristic foraging sites of *P. montanus* in the inner tree parts (Alatalo and Moreno, 1987).

Because competitors are absent from Gotland, *P. ater* has access to a wider food base, which has enabled it to increase its population density and make up numerically for the two absent species (Alerstam et al., 1974; Alatalo et al., 1986). This probably leads to enhanced intraspecific interference competition with concomitant selection for large body size. And the takeover by *P. ater* of the foraging sites of the absentees entails adoption of their easier and less demanding locomotion modes. Selection for manoeuvrability and a small body size is thus relaxed when *P. ater* is geographically isolated from *P. cristatus* and *P. montanus*. Therefore, any evolutionary shift in body size, which might occur in *P. ater* because of relaxed interspecific competition and ensuing niche enlargement, should be towards a larger size.

Here we test this *a priori* hypothesis by comparing 11 morphological measurements that we collected from living specimens of *P. ater* on the Swedish mainland and on six islands where *P. cristatus* and *P. montanus* are naturally absent. The island populations serve as six independent replicates.

The results reported here are based on fieldwork that we performed in the four consecutive years 1979–1982. A study, done subsequent to the completion of our fieldwork, compared the size of a single trait – the tarsus length – of *P. ater* between the island of Gotland and an area near Uppsala on the mainland of east-central Sweden (Alatalo and Gustafsson, 1988). Birds on Gotland had longer tarsi than mainland birds and the large size was shown to be genetically determined.

## HYPOTHESIS AND PREDICTIONS

Among the three *Parus* species that coexist in coniferous forests in Scandinavia, *P. ater* (9.4 g) is the smallest, *P. cristatus* (11.0 g) comes next and *P. montanus* (11.5 g) is largest (mass of first-year birds from the Swedish mainland; Table 2). Selection for efficiency and energy economy leads to coadaptation between the body size and the specific feeding sites, body postures and locomotion modes used during foraging (Palmgren, 1932; Haftorn, 1956c; Lack, 1971; Norberg, 1979; Moreno and Carrascal, 1993). Locomotor agility and flight manoeuvrability show a strong inverse relationship with body size (Andersson and Norberg, 1981). A small body size will therefore be more strongly selected for the more often that mechanically demanding foraging postures and energetically expensive locomotion modes are used during foraging. In places where *P. ater* is geographically isolated from its larger congeneric competitors, it has taken over their more accessible foraging sites in trees and adopted their less demanding foraging behaviour (Alerstam, et al., 1974; Alatalo, et al., 1985: Gustafsson 1988). The new selection regime should shift the balance between opposing selection forces towards a larger body size.

**Table 2.**
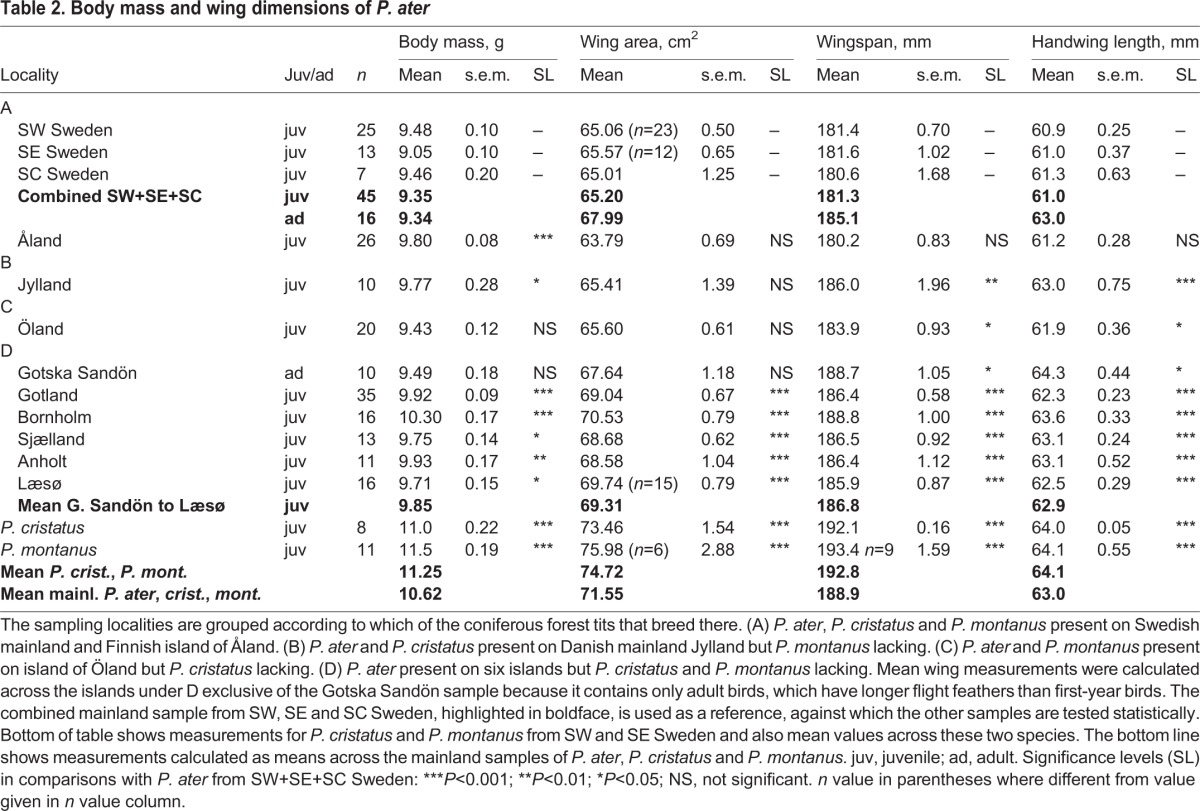
**Body mass and wing dimensions of *P. ater***

In suitable habitat on the Swedish mainland, the population density is about 14 individuals per km^2^ for *P. ater*, 10 for *P. cristatus* and 16 for *P. montanus* (Ulfstrand and Högstedt, 1976; SOF, 1990). So the ecological space per unit forest area is about equally wide for each species. In geographic isolation on islands, *P. ater* therefore has access to three similarly sized niche spaces, two of which are those of the larger absentees. *Our first a priori prediction is that on all six islands, from which* P. cristatus *and* P. montanus *are absent,* P. ater *is larger than its conspecifics on the Swedish mainland and near the average size as taken across* P. ater*,* P. cristatus *and* P. montanus *in sympatry on the Swedish mainland.*

In two other places, *P. ater* co-occurs with one coniferous forest tit – with *P. montanus* on the Swedish island of Öland and with *P. cristatus* on the Danish mainland Jylland. But we have no information about population densities of *P. ater* or its co-existing congener either on Öland or Jylland, except that *P. montanus* occurs ‘scantily’ on Öland (SOF, 1990; Kullberg and Ekman, 2000). Therefore, we cannot estimate what share of the single vacant resource that *P. ater* might procure. But because the vacant niche space is smaller than on the six islands where *P. ater* is alone, selection for a larger body size, suited to exploit the widened niche, should be relatively weak. *Our second prediction therefore is that* P. ater *has increased in size also in these two localities but not as much as on the six islands where it is isolated from both* P. cristatus *and* P. montanus*.*

We included the Finnish island of Åland, located in the Baltic Sea, as a control island. It was colonized by *P. cristatus* as late as in the 1930s (Haila et al., 1979) and now stands out from the other islands by harbouring all three coniferous forest tit species – like the Swedish mainland. *Our third prediction is that* P. ater *is the same small size on the island of Åland as on the Swedish mainland.*

## RESULTS AND DISCUSSION

Among the three *P. ater* samples from the Swedish mainland, the one from South West (SW) Sweden is the largest and contains 25 juvenile and 11 adult birds. We will use that as a reference against which we contrast measurements from each of the other sampling places.

**Fig. 2.**
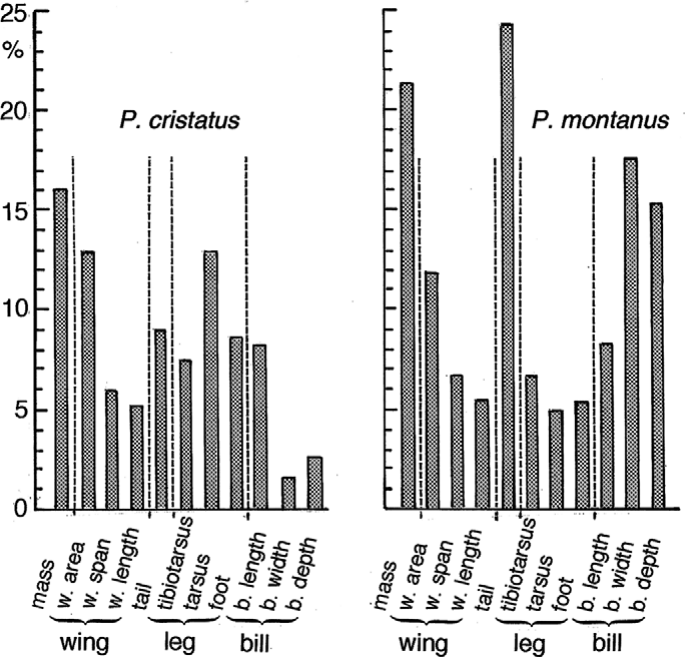
**Diagrams showing by how many percent each of 11 size measures of *P. montanus* and *P. cristatus* from SW and SE Sweden exceeds the size of *P. ater* in the reference sample from SW Sweden.** Tarsus refers to tarsus ‘short’. The diagrams are directly comparable to those in Fig. 3, where the size of *P. ater* from each of 11 sampling places is also compared with the *P. ater* reference sample from SW Sweden.

**Fig. 3.**
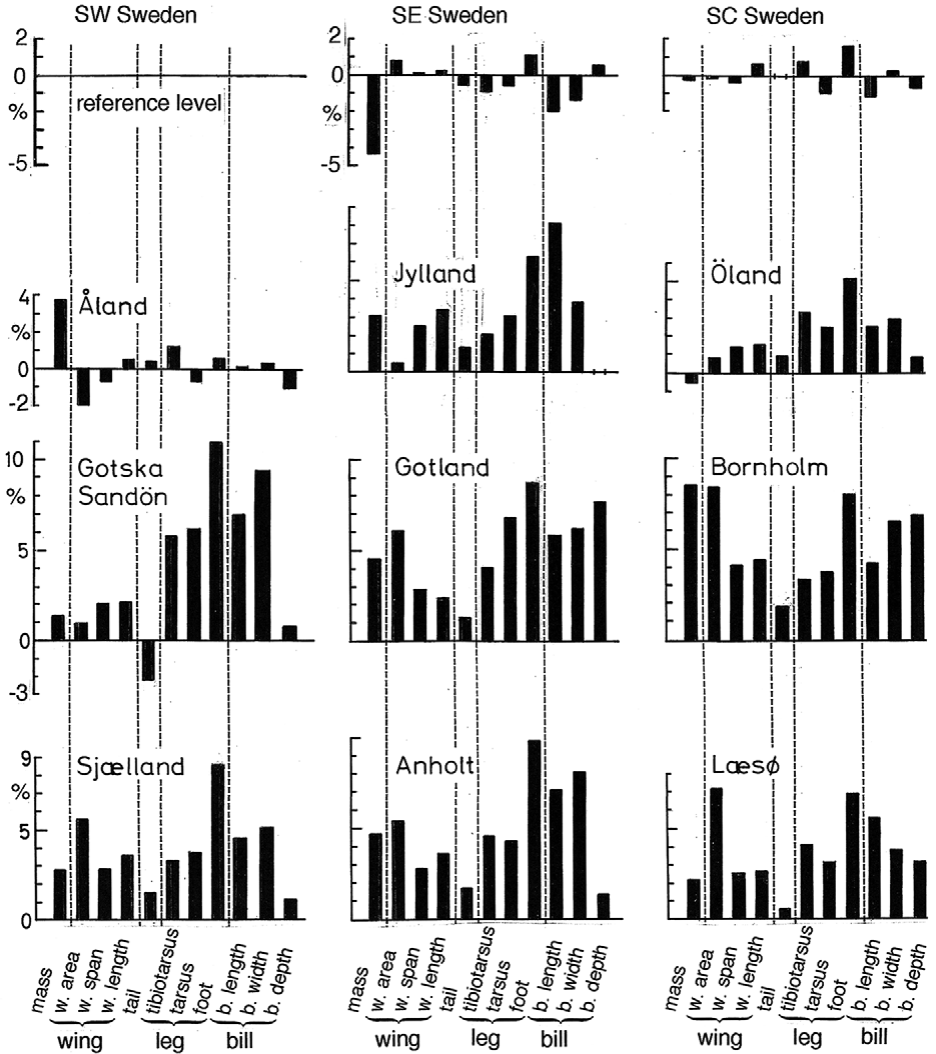
**Diagrams showing by how many percent each of 11 size measures of *P. ater* from 11 sampling places differs from the *P. ater* reference sample from SW Sweden.** Tarsus refers to tarsus ‘short’. *P. ater* coexists with *P. cristatus* and *P. montanus* on the Swedish mainland and on the Finnish island of Åland. On the Danish penisnsula Jylland it coexists with *P. cristatus* and on the island of Öland with *P. montanus.* But on the six islands Gotska Sandön, Gotland, Bornholm, Sjælland, Anholt and Læsø *P. cristatus* as well as *P. montanus* are totally lacking.

First, we compare measurements of *P. cristatus* and *P. montanus* from the Swedish mainland with one another and with *P. ater* from the mainland of SW Sweden. Second, we compare *P. ater* from SW Sweden with conspecifics from each of the other 11 capture sites, two of which lie on the Swedish mainland. This comparison shows the degree of variation between the three mainland samples and gives a quick overview of differences between mainland and island birds (Figs 1-3).

Measurements of *P. ater* turn out to be very similar between SW, South East (SE) and South Central (SC) Sweden. Therefore, and because those birds belong to the same cohesive population, we pool the three mainland samples. Each of the nine offshore samples of *P. ater* is then tested statistically against the unified mainland sample (Tables 2-5). Finally, we compare the body shape of *P. cristatus*, *P. montanus* and *P. ater* from the Swedish mainland with *P. ater* from each of the six islands where *P. cristatus* and *P. montanus* are naturally absent (Table 5).

**Table 3.**
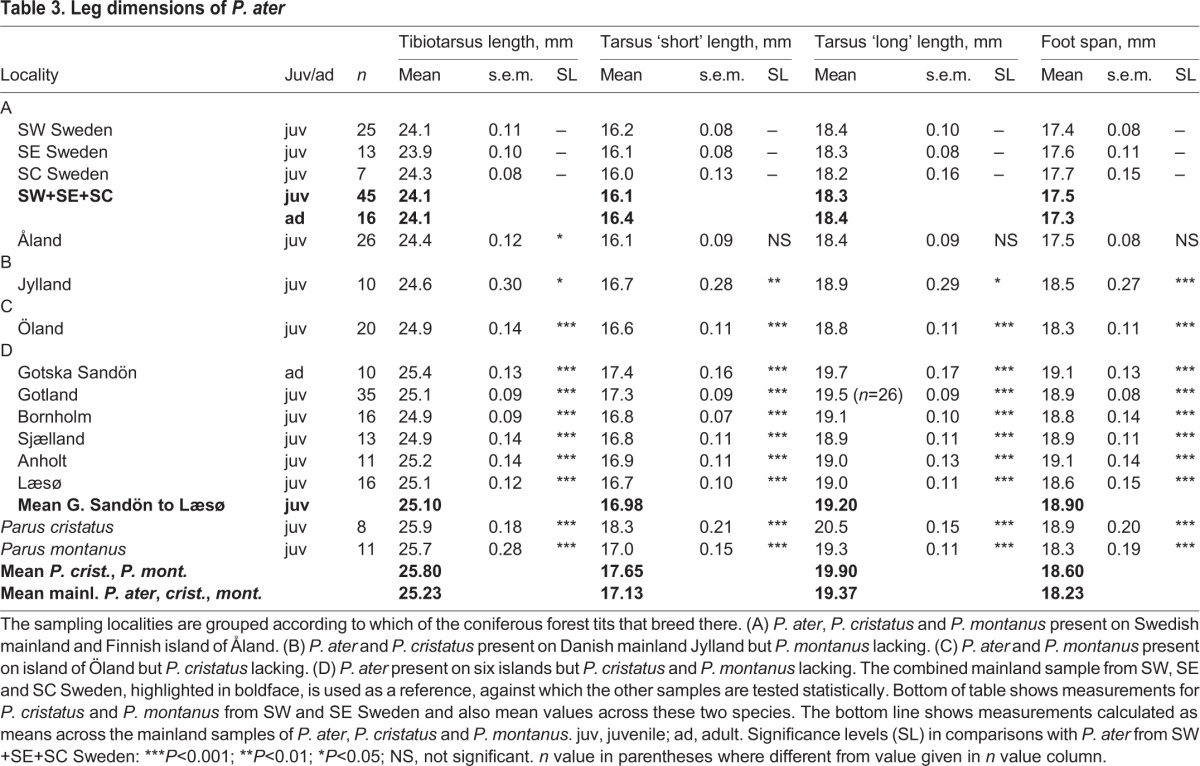
**Leg dimensions of *P. ater***

**Table 4.**
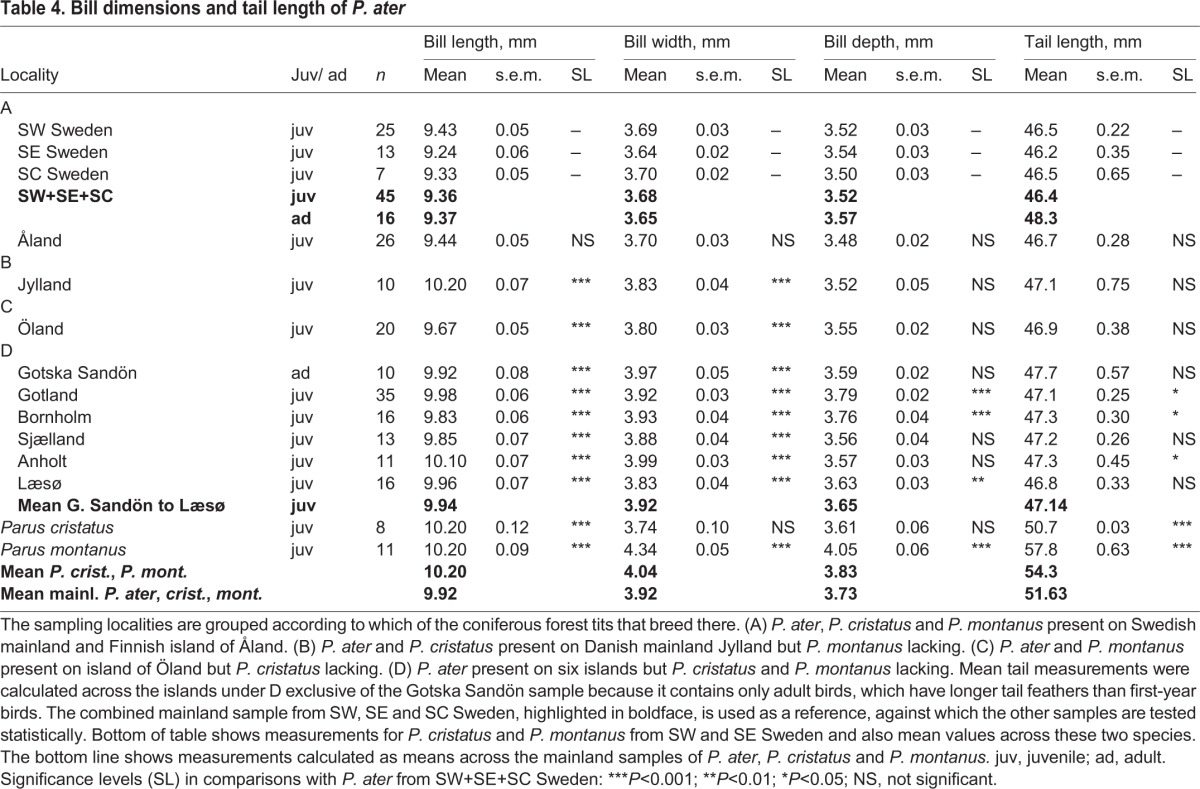
**Bill dimensions and tail length of *P. ater***

**Table 5.**
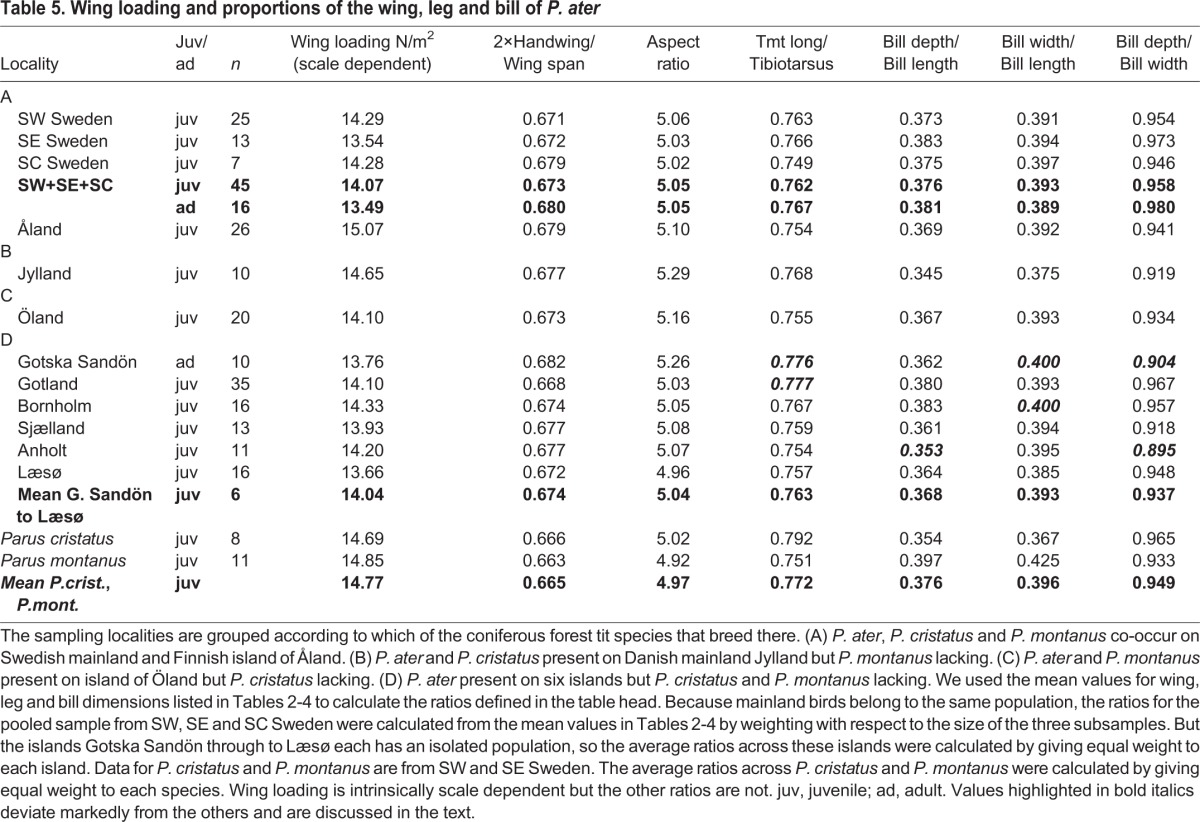
**Wing loading and proportions of the wing, leg and bill of *P. ater***

### Body size of *P. ater*, *P. cristatus* and *P. montanus* in sympatry on the Swedish mainland

Actual measurements of *P. ater*, *P. cristatus* and *P. montanus* from the Swedish mainland, and of *P. ater* from islands, are shown in Tables 2-4. And Fig. 1, inset at top left, shows how three compound measures of the wing, leg and bill of *P. cristatus* and *P. montanus* from SW and SE Sweden differ from the reference sample of *P. ater* from SW Sweden. Likewise, Fig. 2 shows how each of 11 morphological measurements of *P. cristatus* and *P. montanus* from SW and SE Sweden differs from the reference values for *P. ater* in SW Sweden.

*P. cristatus* and *P. montanus* are fairly similar in overall size. But *P. cristatus* has 7% longer tarsus and 3% longer foot than *P. montanus*. Their bills are equally long, but *P. montanus* exceeds the size of *P. cristatus* by 16% in bill width, 12% in bill depth and 14% in tail length.

Both *P. cristatus* and *P. montanus* are considerably larger than *P. ater* from the Swedish mainland. The difference from *P. ater* is statistically highly significant in all measurements except for the depth and width of the bill of *P. cristatus* (Tables 2-4). Notice that Figs 1 and 2 show how much *P. cristatus* and *P. montanus* from the Swedish mainland differs from *P. ater* in SW Sweden whereas the actual measurements appear at the bottom of Tables 2-4.

### Body size of *P. ater* from SW Sweden compared with conspecifics from each of 11 other sampling places

In order to see how the body size of *P. ater* varies between the three mainland samples and whether island birds differ from them, we use the SW Swedish measurements as reference values, against which we contrast data from each of the 11 remaining sampling places. Two of them lie on the Swedish mainland (SC South-Central and SE Sweden), one lies on the Danish mainland Jylland and eight are on different islands.

Fig. 1 gives a quick overview of the results. The bar diagrams show how much birds at the respective locality differ in linear dimensions from birds in SW Sweden. Each bar shows the difference as averaged across three primary measurements of the wing, leg and bill, respectively.

The difference in wing dimensions, shown by the left bar W, was calculated by first finding the amount, expressed in per cent of the SW Swedish value, by which birds from the respective locality differs from the SW Swedish sample in each of the three wing measurements (wing area)^0.5^, wing span and hand-wing length. We then took the average value of these three percentages. Similarly, the local deviation from the SW Swedish measurements of the leg, shown by the middle bar L, is the average of the percentage differences in tibiotarsus length, tarsus ‘short’ length and foot length. And the local deviation from the SW Swedish measurements of the bill, shown by the right bar B, is the average of the percentage differences in bill length, width and height.

Fig. 3 is similar to Fig. 1. But while each bar in Fig. 1 represents the average difference across three measurements, Fig. 3 shows how much each one of 11 morphological measurements of *P. ater* from each of 11 sampling places differs from the reference sample from SW Sweden.

The measurements are very similar between the three sampling places on the Swedish mainland (Tables 2-4; Figs 1, 3). And on the island of Åland, where *P. ater*, *P. cristatus and P. montanus* coexist – like on the Swedish mainland – *P. ater* is the same small size as conspecifics on the SW Swedish mainland (Fig. 1, top right; Fig. 3, left diagram in second row). But on all six islands from which both *P. cristatus* and *P. montanus* are absent, *P. ater* is significantly larger in wing, leg and bill dimensions than its conspecifics on the SW Swedish mainland. Compared with conspecifics in SW Sweden, *P. ater* is also larger on the Danish mainland Jylland, where it coexists with *P. cristatus*, and on the island of Öland, where it coexists with *P. montanus* (Figs 1, 3).

### Body size of *P. ater* in the pooled mainland sample compared with conspecifics from each of nine offshore places

Among all measurements of *P. ater* in the samples from SW, SE and SC Sweden, it is only the body weight of birds in SE Sweden that differs statistically by being about 4.4% lower than in the SW and SC Swedish samples (*P*<0.02). But because body mass varies with food supply and time of day – as detailed below – a mass difference of this magnitude may not be a reliable measure of overall body size.

Apart from this deviation, there is great homogeneity among all the other measurements of *P. ater* from the Swedish mainland (Tables 2-4; Fig. 3). Therefore, and because mainland birds belong to the same uniform and cohesive population, we added together the samples from SW, SE and SC Sweden into a combined mainland sample consisting of 45 juvenile and 16 adult birds. Each measurement in the pooled mainland sample was obtained by averaging across the three sub-samples, weighted in proportion to their respective size. The nine samples from localities off the Swedish mainland were then tested statistically, one at a time, against this unified mainland sample (Mann–Whitney *U*-test). The statistical analyses are based on juvenile birds except for the Gotska Sandön sample, which only contains adults that we compare with adults from the mainland.

This comparison differs from the previous one (Figs 1, 3) insofar as here we contrast each sample collected off the Swedish mainland against the pooled mainland sample rather than against the sub-sample from SW Sweden. The results are reviewed below and shown separately for each measurement in Tables 2-4 and Fig. 3.

### *P. ater* in sympatry with *P. cristatus* and *P. montanus* on the island of Åland

The island of Åland is included as a control because it is comparable to the other six islands except that it harbours all three tit species – like the Swedish mainland. Among 12 measurements of *P. ater*, the body mass and tibiotarsus length are significantly larger on Åland than on the Swedish mainland (Tables 2-4).

On the mainland, the body mass averages 9.35 g for juveniles and 9.34 g for adults, whereas juveniles on the Åland island weigh 9.80 g and adults 9.46 g (*n*=5). This is 0.45 g and 4.8% respectively 0.12 g and 1.3% more than for mainland birds. In general, such small differences in body mass may not be reliable measures of body size because they can be due to different feeding conditions and different times of day for weighing. In a study in Norway, the daily mass increase of *P. ater* amounted to 9.8-10.6% (Haftorn, 1992), which is twice the difference between mainland and Åland birds. As noted above, body mass seems to be decoupled from the other size measures also among *P. ater* from SE Sweden (Fig. 3; Tables 2-4).

The difference in tibiotarsus length is small. On Åland, the average length is 24.4 mm for juveniles and 23.9 mm for adults (*n*=5) vs 24.1 mm both for juveniles and adults on the Swedish mainland. So the tibiotarsus is actually shorter in adults on Åland. And wing area, wingspan and bill depth are smaller in juvenile birds on Åland than on the Swedish mainland, whereas the other measurements are very similar between Åland and the Swedish mainland (Fig. 2; Tables 2-4).

Taken together, the measurements show that *P. ater* is the same small size on Åland as on the Swedish mainland (Tables 2-4; Figs 1, 3). This is expected since *P. ater* coexists with *P. cristatus* and *P. montanus* on Åland – like on the Swedish mainland – entailing similar interspecific competition.

### *P. ater* in geographical isolation from *P. cristatus* and *P. montanus* on six islands

*P. ater* is substantially larger on each of the six islands Gotska Sandön, Gotland, Bornholm, Sjælland, Anholt and Læsø – all of which lack *P. cristatu*s and *P. montanus –* than on the Swedish mainland, where *P. ater* coexists with *P. cristatu*s and *P. montanus.* The amount by which *P. ater* on the islands differs from mainland conspecifics varies between body parts and also between the islands (Fig. 3). The maximum size difference recorded for a single trait between island and mainland first-year birds is 8% for a wing dimension, 10% for a leg dimension and 9% for a bill dimension (Fig. 3; Tables 2-4).

Each of the six independent island-mainland comparisons involves 12 measures of body size (Tables 2-4; Fig. 3, showing 11 measurements because tarsus ‘long’ is omitted). Of the resulting 72 comparisons of primary measurements between island and mainland birds, 56 differ in the expected direction at the 0.001 significance level, two at the 0.01 level and six at the 0.05 level, whereas eight comparisons do not reach statistical significance. The latter are body mass, wing area, bill depth and tail length of adult birds on Gotska Sandön; bill depth and tail length of juveniles on Jylland; bill depth of juveniles on Anholt; and tail length of juveniles on Læsø. It is noteworthy that there is not a single local mean measurement of *P. ater* that is significantly smaller on any of the six islands than on the Swedish mainland (Tables 2-4; Fig. 3).

Because each measurement is a measure of body size, the various dimensions of an individual bird are strongly correlated. Taken together, however, the data firmly establish that the overall body size of *P. ater* is significantly larger on the islands than on the Swedish mainland. As an additional point, the probability that a size change would occur towards larger birds on all six islands independently, and due to random events only, is 0.5^6^=0.016, a significant result in itself.

Tables 2-5 show measurements calculated as means across the six islands Gotska Sandön through to Læsø, all of which entirely lack *P. cristatus* and *P. montanus.* Each of these islands has an isolated *P. ater* population of its own, so the means are calculated by giving equal weight to each island.

The bottom of Tables 2-5 shows measurements for *P. cristatus* and *P. montanus* from SW and SE Sweden and also mean values across these two species. And the bottom line in Tables 2-4 shows measurements calculated as means across mainland *P. ater*, *P. cristatus* and *P. montanus*. The reason for calculating average values across *P. cristatus* and *P. montanus* as well as across *P. ater*, *P. cristatus* and *P. montanus* in sympatry on the Swedish mainland is that natural selection might drive *P. ater* in those directions on islands where *P. cristatus* and *P. montanus* are lacking.

Several of the measurements of *P. ater*, calculated as means across the six islands G. Sandön through to Læsø, are very similar to the three-tit means, calculated across *P. ater*, *P. cristatus* and *P. montanus* in sympatry on the Swedish mainland. The respective means are: 62.9 vs 63.0 mm for the hand-wing; 25.10 vs 25.23 for tibiotarsus; 16.98 vs 17.13 for tarsus ‘short’; 9.94 vs 9.92 for bill length; 3.92 vs 3.92 for bill width; and 3.65 vs 3.73 for bill depth (Tables 2-4). So, on the six islands, from which *P. cristatus* and *P. montanus* are totally lacking, *P. ater* has increased in size and is near the mean size as calculated across *P. ater*, *P. cristatus* and *P. montanus* in sympatry on the Swedish mainland.

Another study, performed three years after we had completed our fieldwork, compared the size of *P. ater* between the island of Gotland and an area near Uppsala on the mainland of east-central Sweden. The only dimension measured was the length of tarsus. It was 5.9% longer in adult males (mean 19.13 mm) and 5.6% longer in adult females (mean 18.72 mm) on the island of Gotland than on the mainland (Alatalo and Gustafsson, 1988). These results are in good agreement with the differences that we recorded for the ‘long’ tarsus of first-year birds (not separated by sex). In our Table 3, the tarsus length of first-year birds on Gotland exceeds that of mainland birds from SW, SE and SC Sweden by 6.0%, 6.6% and 7.1%, respectively.

### *P. ater* in sympatry with one competitor in two places

*P. cristatus* co-occurs with *P. ater* on the Danish peninsula Jylland and *P. montanus* co-occurs with *P. ater* on the island of Öland. But we have no data on the population density of *P. cristatus* on Jylland or of *P. montanus* on Öland, except that *P. montanus* occurs scantily on Öland (SOF, 1990; Kullberg and Ekman, 2000). So we do not know how strong interspecific competition that *P. ater* is exposed to in these two places.

In the absence of only one of the two larger coniferous forest congeners, *P. ater* is nonetheless larger in both places than on the Swedish mainland where it coexists with both *P. cristatus* and *P. montanus* (Figs 1, 3; Tables 2-4). The comparison of *P. ater* from the Swedish mainland with conspecifics from Jylland and Öland involves 12 measures of body size. Of the resulting 24 comparisons, 17 show statistically significant differences in the expected direction, whereas 7 do not. The latter are wing area, bill depth and tail length of *P. ater* on Jylland; and body mass, wing area, bill depth and tail length of *P. ater* on Öland. But there is not a single local mean measurement of *P. ater* that is smaller in any of these two places than on the Swedish mainland (Tables 2-4; Fig. 3).

Neither in Jylland nor on Öland does the overall size of *P. ater* reach the size attained on any of the six islands, from which both *P. cristatus* and *P. montanus* are absent. This is as predicted because the niche space of *P. ater* is narrower on Öland and Jylland, both places of which offer only one vacant niche – that of one absentee – that may be divided up by the two species present. But on the six islands, where *P. ater* is alone, there are two vacant foraging niches – those of the two absent congeners – that can be added in their entirety to the foraging space of *P. ater*. Inclusion by *P. ater* of the foraging sites of the absentees entails adoption of their less demanding locomotion modes, which relaxes selection for manoeuvrability and a small size.

The probability is 0.5^8^=0.004 that a size change would occur towards larger birds independently, and at random, on all six islands where *P. ater* is alone, as well as on Jylland and Öland where one competing congener is lacking. And except for the change of body size in the predicted direction in all eight places, where it was expected to happen, also the magnitude of the size increase is as anticipated.

### Variability

Small populations tend to show reduced genetic and phenotypic variability due to accidental loss of alleles by genetic drift (Mayr, 1965; Grant and Grant, 2008). The populations studied here differ enormously in size. Anholt is the smallest island and based on the coniferous forest size we estimate its *P. ater* population to be 200 individuals whereas Gotland, which is the largest island, harbours 80,000 (SOF, 1990) and the Swedish mainland 2 million individuals (Ulfstrand and Högstedt, 1976).

The coefficient of variation, defined as the standard deviation expressed in per cent of the mean, was calculated for all measurements of *P. ater* listed in Tables 2-4. It mostly lies between 2 and 4%. The variance of measurements in the sample from SW Sweden was used as a reference against which we tested the variance of each of the other samples (variance-ratio *F*-test). There is no trend in the size of the coefficient of variation between island and mainland birds or between small and large populations. In particular, the coefficients of variation in the very small population of *P. ater* on the island of Anholt are not any different from the large populations on Gotland and the Swedish mainland. So, the small Anholt population seems not to have lost phenotypic variability.

### Body shape of *P. ater*, *P. cristatus* and *P. montanus* from the Swedish mainland compared with *P. ater* from six islands

Body shape is described here in terms of ratios between various measurements in Tables 2-4. Seven ratios, defined in Table 5, were calculated for each of the 12 samples. We also calculated the average value of each ratio across the three mainland samples from SW, SE and SC Sweden. Because mainland birds belong to the same uniform population, we weighted the sub-samples in relation to their size. But populations on each of the six islands Gotska Sandön through to Læsø are separate entities. Therefore, we calculated the average ratios across these islands by giving equal weight to each island. The average ratios are set in boldface in Table 5.

In the absence of *P. cristatus* and *P. montanus* from the islands, *P. ater* might be expected to evolve morphologically in the direction of one or the other of the missing congeners, or towards some average of their respective shapes. Therefore, we also present ratios as averaged across *P. cristatus* and *P. montanus* from the Swedish mainland, using data from Tables 2-4 and giving equal weight to each species. These across-species average ratios are set in boldface at the bottom of Table 5.

First and foremost, there are only small differences in the shape and proportions of the wing, leg and bill between *P. ater*, *P. cristatus* and *P. montanus* on the Swedish mainland. Therefore, one would not expect any large changes in body proportions of *P. ater* in places where *P. cristatus* and *P. montanus* are lacking. And overall, the body proportions of *P. ater* are rather similar between the islands and the Swedish mainland. However, in a few cases, set out in bold italics in Table 5 and reviewed below, the shape of *P. ater* differs between the islands and the Swedish mainland.

Wing loading is equal to (body weight)/(wing area) and is proportional to *l*^3^/*l^2^*∝*l* under geometric similarity, *l* being a representative length. So, wing loading is scale dependent and varies in proportion to the linear dimension among different sized animals of the same shape. The wing loading is 4.4% larger in *P. cristatus* and 5.5% larger in *P. montanus* than in *P. ater* in the pooled mainland sample (Table 5). But the linear wing dimensions of *P. cristatus* and *P. montanus* exceed those of *P. ater* by about the same percentages as the wing loading (Fig. 2). Therefore, the higher wing loading in *P. cristatus* and *P. montanus* is largely a scale effect of their larger body size. But it is noteworthy that even though *P. ater* is larger on the islands than on the mainland the wing loading is not higher in island birds, so in this respect there is no convergence on *P. cristatus* and *P. montanus* (Table 5).

The proportion that the hand-wing makes up of the wing span is 1% smaller in *P. cristatus* and 1.5% smaller in *P. montanus* than in mainland *P. ater*, but it is identical between mainland and island *P. ater.* And the wing aspect ratio is similar between *P. cristatus* and mainland *P. ater*, whereas it is 2.6% smaller in *P. montanus* than in mainland *P. ater*. But the aspect ratio of *P. ater*, as averaged across the six islands, is equal to that of mainland *P. ater.* So, wing shape of *P. ater* has not changed on the islands.

The ratio between tarsus and tibiotarsus is 1–3.3% larger on the islands of Gotska Sandön (ad, 0.776) and Gotland (juv, 0.777) than in mainland conspecifics (ad, 0.767; juv 0.762), so on these islands *P. ater* approaches the leg shape of *P. cristatus* (Table 5). But this is not a general trend for the islands; the tarsus/tibiotarsus ratio of *P. ater* as averaged across all six islands, from Gotska Sandön through to Læsø, is 0.763 for juveniles (*n*=91) and 0.767 for adults (*n*=27). This is in close agreement with the ratios 0.762 and 0.767, respectively, for the unified mainland sample (Table 5). What is more, the tarsus/tibiotarsus ratio of mainland *P. ater* is similar to the mean ratio taken across *P. cristatus* and *P. montanus* in allopatry on the Swedish mainland. Therefore, there should not be any directional selection on leg proportions in *P. ater* when *P. cristatus* and *P. montanus* are absent.

The bill is the same length in *P. cristatus* and *P. montanus* (Table 4). But it is stouter in *P. montanus*, being both deeper and wider and in both respects differs from *P. cristatus* at the 0.001 significance level. As a result, the bill's depth/length and width/length ratios are larger in *P. montanus* than in *P. cristatus*. In *P. ater* from the Swedish mainland, both ratios are halfway between those of *P montanus* and *P. cristatus* (Table 5). So, when each of the depth/length and width/length bill ratios is averaged across *P. cristatus* and *P. montanus*, the mean across-species ratios are 0.376 and 0.396, respectively, which is strikingly similar to the corresponding ratios 0.376 and 0.393 of *P. ater* on the mainland (Table 5). Therefore, there is no obvious direction in which bill shape of *P. ater* might be expected to evolve on islands in the absence of *P. cristatus* and *P. montanus*.

But there are a few local deviations in bill shape. On the island of Anholt, the depth/length ratio of the bill of *P. ater* stands out by being 6.1% lower in juveniles and 8.1% lower in adults than in mainland conspecifics. In this respect, *P. ater* on Anholt has converged on the bill shape of mainland *P. cristatus* to the extent that they are almost identical; the bill's depth/length ratio of *P. ater* on Anholt is 0.353 in juveniles (*n*=11) and 0.350 in adults (*n*=3) vs 0.354 (*n*=8) and 0.360 (*n*=3), respectively, in *P. cristatus* on the mainland (Table 5). This low depth/length ratio is due entirely to a small bill depth in *P. ater* on Anholt (Table 4).

Overall, the bill's depth/length ratio of *P. ater*, as averaged across all six islands (juv 0.368; ad 0.366), is slightly lower than the average for mainland conspecifics (juv 0.376; ad 0.381). This is a general deviation on islands in the direction of *P. cristatus*.

On Gotska Sandön and Bornholm, *P. ater* has proportionately wide bills (width/length ratio=0.400), a deviation toward the bill shape of *P. montanus* (width/length ratio=0.425). But the width/length ratio for *P. ater*, again averaged across the six islands (juv 0.393; ad 0.389), is exactly identical to the average for mainland *P. ater* (juv 0.393; ad 0.389) and very similar to the average ratio taken across *P. cristatus* and *P. montanus* (juv 0.396; ad 0.388) (Table 5).

The depth/width ratio of the bill of *P. ater* is 6.6% lower in juveniles from Anholt (0.895) and 7.8% lower in adults from Gotska Sandön (0.904) than in conspecifics on the Swedish mainland (0.958 for juveniles and 0.980 for adults). These island deviations are in the direction of *P. montanus* (0.933).

There are thus occasional differences in body proportions of *P. ater* between some of the islands and the Swedish mainland. But there is no general trend, common to all islands, towards the shape of one or the other of the two absent species. Most of the ratios of *P. ater*, as averaged across the six islands Gotska Sandön through to Læsø, are almost identical to the corresponding ratios of mainland conspecifics (Table 5; average ratios in boldface).

In conclusion, the occasional differences in body shape of *P. ater* between some of the islands and the mainland occur variously in the direction of one or the other of the two absent congeners. The lack of consistency between islands suggests the deviations are due to fortuitous founder differences or random drift. But the shape differences could also be the result of evolutionary adaptation in different directions on different islands owing to habitat differences.

### The large body size of *P. ater* on islands is genetically determined

Two cross-fostering experiments have been done to find out whether the large size of *P. ater* on the islands has a genetic basis. The switch occurred at the egg-stage and involved entire clutches in both experiments. In the first case, eggs were exchanged between nests on the island of Anholt and nests in an area 50 km east of Gothenburg in south-western Sweden (R.Å.N. and Raimo Neergaard-Thörgersen, unpublished). In the other, eggs were exchanged between nests in the southern part of the island of Gotland and nests in an area near the city of Uppsala in east-central Sweden (Alatalo and Gustafsson, 1988). Both experiments show conclusively that the enlarged body size of *P. ater* on the islands is genetically determined and not due to environmental effects on the phenotype.

### Adaptation in island *P. ater* in the presence of gene flow

**Fig. 4.**
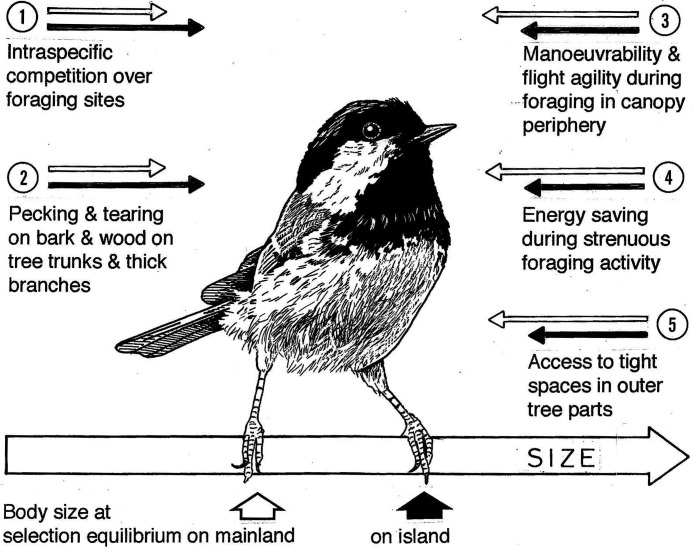
**Ecological selection forces acting on the body size of *P. ater*.** Open arrows symbolize selection pressures on *P. ater* when *P. cristatus* and *P. montanus* are present, solid arrows when they are absent. Arrow length in each pair indicates presumed relative selection strengths in sympatry and allopatry, respectively. Conditions on the left side act to increase body size and they gain in importance in allopatry on the islands. Those on the right side act to reduce body size and they lose significance on the islands. When *P. cristatus* and *P. montanus* are absent, *P. ater* takes over the vacant space, which creates a new selection regime. The widened food base permits a higher population density. It probably increases intraspecific, interference competition, which in turn selects for a large body size. And the take-over of foraging sites on the inner parts of branches and on the trunk, normally occupied by *P. cristatus* and *P. montanus*, gives more opportunity for obtaining food by pecking and tearing, which selects for a large size. In sympatry on the mainland, *P. ater* forages mainly in the canopy periphery, which requires high manoeuvrability and is expensive in energy. A low body mass improves manoeuvrability and reduces energy costs for locomotion and, in addition, facilitates access to tight spaces in the canopy periphery. The frequent use of outer branch parts by *P. ater* on the mainland therefore selects for a small body size. But when *P. ater* exploits the foraging sites of the absentees on the islands, it cuts down on its use of the canopy periphery, which relaxes selection for a small body size. Conflicting selection forces therefore cancel out at a larger body size of *P. ater* on the islands than on the mainland where it coexists with *P. cristatus* and *P. montanus*.

Body size is determined by several selection forces, some of which act in opposite directions (Fig. 4). The direction and strength of selection are determined by environmental conditions and behaviour and depend on the current body size. When body size has reached its optimal value, the conflicting selection forces balance out. But if conditions are different in a newly colonized area, directional selection sets in and acts to shift body size towards a new optimum. While a trait is in the process of responding to a recent selection force, the gap becomes progressively narrower between the current state and the optimum. But the strength of directional selection diminishes as the phenotype nears its optimal state, which is therefore approached asymptotically and ever more slowly.

Adaptive evolution on the islands will also be slowed by gene flow from the mainland population due to the occasional arrival of immigrants and ensuing interbreeding events. And adaptation comes to an end when the strength of directional selection has dropped to the level where it is offset by the rate of inflow of those genes that are selected against. In the presence of gene flow, adaptation therefore ceases before the optimal state is reached. The higher the rate of gene flow the wider the gap between the attainable character state and the optimal state. For these reasons, the locally optimal body size of *P. ater* may not be attained on the islands.

*P. ater* is a permanent resident the whole year through within its range on the mainland of Norway, Sweden and Finland as well as on the islands. But it occasionally performs irruptive migrations. In the 41-year period from 1973 to 2013, an unusually large autumn irruption occurred in 1975, when 15,515 individuals of *P. ater* were recorded on migration at Falsterbo Bird Observatory on the SW-most point of Sweden (Fig. 1). Smaller autumn irruptions occurred in the following years, with bird counts in parentheses: 1982 (518), 1986 (277), 1988 (2175), 1990 (4479), 1991 (1976), 1993 (504), 1996 (129), 2003 (880), 2008 (320) and 2012 (533). In the other years, only a few migrating birds were recorded and in 15 of the 41 years there was not a single record of *P. ater* at Falsterbo Bird Observatory, from which these migration counts were obtained.

Judging from ringing data from Scandinavian bird observatories, migrating *P. ater* stay inland of the west and east coastlines of Sweden (Ottenby Report, 1989). Therefore, and because irruptions are infrequent, gene flow is probably weak to the most isolated islands Gotska Sandön, Gotland, Bornholm, Anholt and Læsø.

### Selection for a large body size in *P. ater* when *P. cristatus* and *P. montanus* are absent

We will now examine how the absence of *P. cristatus* and *P. montanus* from the islands influences niche width and foraging behaviour of *P. ater* and the selection acting on it. Fig. 4 shows some ecological factors that are likely to determine the difference in body size of *P. ater* between the mainland and islands. We distinguish between forces that select for a large size – acting rightwards in Fig. 4 – and those that select for a small size – acting leftwards – and evaluate their relative strengths on the mainland and the islands.

The population density of *P. ater* has been determined on the island of Gotland, where *P. cristatus* and *P. montanus* are naturally absent. In the winter months December-February, *P. ater* occurred at 5-15 times higher densities in forests of *Pinus silvestris* on Gotland than in forests dominated by *Picea abies* on the mainland of southern Sweden (Alerstam et al., 1974). The high population density of *P. ater* on Gotland was confirmed in a later study. In the winter months January-February, there were 90-110 individuals per km^2^ on Gotland vs 20 per km^2^ on the mainland of east-central Sweden, a difference by a factor 5 (Appendix in Alatalo et al., 1986). *P. ater* thus compensates numerically for the two absent species on Gotland and reaches a population density that is similar to the combined density of *P. ater*, *P. cristatus* and *P. montanus* on the Swedish mainland (Alerstam et al., 1974; Gustafsson, 1988).

On Gotland, *P. ater* has expanded its niche width and exploits the foraging sites used elsewhere by the two absent species (Alerstam et al., 1974; Gustafsson, 1988). And in a mainland study area near the city of Uppsala in east-central Sweden, *P. ater* expanded its foraging niche in a similar way after the number of *P. cristatus* and *P. montanus* had been experimentally reduced by more than 50% (Alatalo et al., 1985).

The niche expansion of *P. ater* enlarges its food base so the associated increase in population density need not necessarily strengthen intraspecific competition for food. But the increased population density likely intensifies behavioural interference competition. This, in turn, should select for a large body size (Fig. 4, point 1).

Body size is also related to feeding site selection and locomotor modes during foraging. *P. cristatus* and *P. montanus* are specialized to forage on the middle and proximal parts of branches and on tree trunks, and their large size is probably an adaptation for this. There are pronounced differences in feeding site selection and foraging behaviour of *P. ater* between, on the one hand, the island of Åland and the Swedish mainland and, on the other hand, the island of Gotland. In coexistence with *P. cristatus* and *P. montanus* on the island of Åland and on the mainland (near the city of Uppsala in east-central Sweden), *P. ater* foraged almost exclusively in the needle-carrying parts of pine trees, spending 90% of its foraging time there and 10% on twigs. In allopatry on Gotland, however, *P. ater* spent only 24% among needled twigs, but 29% on needle-free twigs, 40% on thick parts of branches and 7% on the trunk of *Pinus silvestris* (Fig. 1 and appendix in Alatalo et al., 1986; Fig. 1 in Gustafsson, 1988).

A partial niche takeover occurred also in experimental forest plots on the mainland of east-central Sweden. After the density of *P. cristatus* and *P. montanus* had been experimentally reduced by more than 50%, *P. ater* and *R. regulus* increased their foraging rate in the inner canopy parts, which is the foraging zone of the two population-reduced species (Alatalo et al., 1985). And an aviary experiment, which tested *P. ater* and *P. montanus* against each other, showed that when *P. montanus* tits were removed, *P. ater* turned to exploiting also the characteristic foraging sites of *P. montanus* in the inner tree parts (Alatalo and Moreno, 1987). In all these cases, *P. ater* shifted from foraging predominantly on the peripheral twigs and needles to foraging also on the middle and basal branch parts and on the trunk.

The increased use of the inner tree regions by *P. ater* when *P. cristatus* and *P. montanus* are absent gives more opportunity for obtaining food by pecking and tearing in bark and wood. These activities are favoured by a large body size, which is therefore selected for in *P. ater* on the islands (Fig. 4, point 2).

On the mainland, *P. ater* made frequent short flights in the tree periphery, hovered occasionally and often hung underneath slender and pliant twigs, activities that require good manoeuvrability and are expensive in energy. But on Gotland, it mostly moved by hops on top of branches in an upright position (Appendix in Alatalo et al., 1986; Fig. 1 in Gustafsson, 1988). The shift to foraging more frequently in the inner parts of trees on Gotland is thus associated with a change to easier and less energy-expensive postures and locomotion modes.

Niche differentiation with respect to body size occurs also at the intraspecific level between different-sized individuals of *P. ater*. In isolation from *P. cristatus* and *P. montanus* on Gotland, large individuals of *P. ater* often used the inner parts of trees whereas small individuals foraged more in the needle-carrying outer regions (Gustafsson, 1988). Five size measures – tarsus length, bill length, length of the first secondary wing feather, hand-wing length and tail length – were correlated with body mass, and tarsus length was a particularly reliable measure of body size. The range of size variation was 18.6–20.1 mm for tarsus length and 9.0–11.1 g for body mass (read from Fig. 5B and C in Gustafsson, 1988). So, it was within these limited ranges of variation that a body-size-related niche differentiation was observed among *P. ater* on Gotland. This intraspecific differentiation of feeding sites among different-sized individuals of *P. ater* mirrors the interspecific body-size-related feeding site differentiation that occurs among *P. ater*, *P. cristatus* and *P. montanus* where they coexist on the Swedish mainland. It emphasizes the tight association between body size and foraging zone in trees.

Flight manoeuvrability and general agility increase with decreasing body size for aerodynamic and mechanical reasons (Andersson and Norberg, 1981). And the energy cost of locomotion decreases with decreasing body mass. During short flights within trees, birds often do not attain the maximum-range cruising speed at which energy cost per unit distance is minimized. At slower speeds, energy cost goes up more the lower the speed, peaking at hovering (Pennycuick, 2008). In addition, short flights in tree canopies contain a disproportionately large proportion of energy-expensive manoeuvres, such as take-off and landing and twists and turns for negotiating obstacles in the cluttered canopy space. In an experimental study of *P. montanus*, kept in aviaries that only permitted short flights, the energy cost for flight, measured with the doubly-labelled water technique, was 27–32% higher than that calculated for direct flight at normal cruising speed (Carlson and Moreno, 1992). So, flight within tree canopies is energetically very expensive.

On the mainland, *P. ater* flies more frequently while foraging than do *P. cristatus* and *P. montanus* (Moreno et al., 1988). To see how that affects energy expenditure, the daily field metabolic rate was measured in *P. ater*, *P. cristatus* and *P. montanus* under natural conditions, using the doubly-labelled water technique. The measurements were made on free-ranging birds when temperatures were below freezing in mid-winter (December-January) in a forest near Uppsala in east-central Sweden. The daily metabolic rate turned out to be a higher multiple of the basal metabolic rate in *P. ater* than in *P. cristatus* and *P. montanus.* It was 2.89× BMR for *P. ater* and 2.22× BMR for each of the other two, a difference by a factor 1.3 (Moreno et al., 1988). The difference is obviously a consequence of the more frequent use of energy-demanding locomotion modes, like flight, by *P. ater*.

On the mainland, small size is therefore particularly important to *P. ater* for keeping down energy costs for locomotion (Fig. 4, point 4). But because a lower proportion of the foraging time is spent in the canopy periphery on the islands, selection for a small body size must be weaker there than on the mainland (Fig. 4, point 3-5). In addition, a small size is favoured more when foraging in the tight spaces in the meshwork of twigs and needles in the periphery of the tree canopy than when foraging in less cluttered spaces near the trunk (Fig. 4, point 5).

We conclude that the evolutionary increase in body size of *P. ater* on the islands is due ultimately to the absence of *P. cristatus* and *P. montanus*. It was likely brought about by the combination of intensified selection for a large size, due to increased intraspecific interference competition following niche expansion and population increase, and weakened selection for a small size following a shift to easier foraging modes on the islands (Fig. 4). Other selection forces on body size are discussed under ‘Alternative explanations’. But they act in the opposite direction to the evolutionary change that has actually taken place and therefore cannot explain it. Their effect is obviously overridden by the selection forces reviewed above.

### Interspecific competition among *P. ater*, *P. cristatus* and *P. montanus*

If the large body size of *P. ater* has evolved on the islands because there is no competition from *P. cristatus* and *P. montanus*, then its small size in sympatry with them on the mainland must be due to their presence. Based on the literature, we review evidence for interspecific competition.

*P. ater*, *P. cristatus* and *P. montanus* have similar diet both regarding food items cached in autumn for consumption in winter, as documented in a large study in Norway (Haftorn, 1956c), and food given to nestlings, as recorded in SW Sweden by automatic photography of parents bringing food to their young (von Brömssen and Jansson, 1975). And all three species join up in mixed-species foraging flocks in winter, which is conducive to interspecific competition.

The way population density is regulated has been extensively studied in *P. cristatus* and *P. montanus* but not in *P. ater.* Because the ecology is so similar between these three species, population regulation is probably similar for *P. ater*. Therefore, we refer to results for *P. cristatus* and *P. montanus* in the following. We emphasize field studies done in the Nordic countries because their climate and forest habitat are similar to those of the islands and therefore present similar conditions for population regulation.

Predation by tits causes considerable reduction of their food in winter. In two field experiments in SW Sweden, branches of *Picea abies* were enclosed with nets to prevent birds from foraging on them throughout the winter. In one of the experiments, run in the period October to March, the exclosure reduced spider mortality from 57% on unprotected branches to 34% on protected branches, suggesting that birds consumed 23% of the spiders present in autumn (Askenmo et al., 1977). In the other experiment, the exclosure reduced overwinter mortality of small spiders from 90% on unprotected branches to 75% on protected, indicating that birds consumed 15% of the small spiders present in autumn. For large spiders, mortality was reduced from 85% on unprotected branches to 46% on protected, indicating that birds consumed 39% of the large spiders present in autumn (Table 2 in Gunnarsson, 1983). The higher disappearance rate of spiders from unprotected branches was attributed to predation by *P. ater*, *P. cristatus*, *P. montanus* and *R. regulus*.

Because birds themselves reduce prey density, bird mortality due to starvation acts in a density-dependent way. It has been shown experimentally that energy stress is the main cause of winter mortality among *P. cristatus* and *P. montanus*. After experimental provisioning of extra food in autumn and winter in SW Sweden, winter survival almost doubled during the period November to March, from 39% to 76% for *P. cristatus* and from 45% to 82% for *P. montanus* (Table 4 in Jansson et al., 1981).

At the same study site in SW Sweden, population density of *P. cristatus* and *P. montanus* was experimentally reduced locally in autumn. For *P. montanus*, winter survival in the low-density experimental population increased to 100% whereas survival rate was 68% by April in a normal-density control population. This outcome demonstrates that winter losses are density-dependent and that there is significant intraspecific competition between flock members (Ekman et al., 1981). Another study in SW Sweden, based on year-round monitoring of a population of marked individuals of *P. montanus* over six years, showed that survival is strongly density-dependent among first-year birds in late winter. Mortality in winter is driven by competition for non-renewable food (Ekman, 1984).

*Parus cristatus* and *P. montanus* are considered to be dominant over *P. ater* and to monopolize their species-characteristic feeding sites in the interior of tree canopies, thus preventing *P. ater* from foraging there (Hogstad, 1978; Alatalo and Moreno, 1987; Suhonen et al., 1992, 1993; Suhonen, 1993; but see Haftorn, 1993). There is also some overlap in feeding sites between *P. ater*, *P. cristatus* and *P. montanus*, so in the shared space they reduce each other's food resources directly.

The clearest demonstration that *P. cristatus* and *P. montanus* restrict the foraging niche of *P. ater* comes from the fact that *P. ater* extends it foraging space in their absence. On the mainland, where all three coexist, *P. ater* forages predominantly on the peripheral twigs and needles, but in the absence of *P. cristatus* and *P. montanus* it shifts to foraging also on the middle and basal branch parts and the trunk, places that are normally used by the absentees (see review in the foregoing section on selection for a large body size).

*Regulus regulus* occurs together with *P. ater* in coniferous forests on the Swedish mainland as well as on all the islands, so there is no mainland-island difference in competition between the two (Table 1). They use much the same foraging zones in trees (Haftorn, 1956c) and overlap in diet. *P. ater* eats arthropods and seeds (Haftorn, 1956c) whereas *R. regulus* only eats arthropods all year round, including very small items like Collembola (Thaler, 1973; Hogstad, 1984). *P. ater* is socially dominant over *R. regulus* and often takes over its feeding site by supplanting attacks (R.Å.N., personal observation). *Certhia familiaris* occurs in coniferous forest on the mainland and on all the islands except Anholt and Læsø (Table 1). It forages almost entirely on tree trunks and therefore interferes little with *P. ater*.

In conclusion, food is a limiting factor that brings about population regulation in winter. The process is density-dependent because the rate of food depletion increases with increasing population density and feeds back to the individual in terms of food scarcity. Winter is also the season when *P. ater* occurs together with *P. cristatus* and *P. montanus* in mixed-species foraging flocks, which is conducive to interspecific competition. Regardless of whether *P. cristatus* and *P. montanus* restrict the foraging space of *P. ater* physically, or if they reduce food in their foraging zones to levels at which it is not profitable for *P. ater* to forage, they compete by reducing food accessibility for it. In addition, they reduce food for *P. ater* directly in the overlapping parts of their respective foraging space. Since winter is the season when population regulation mostly takes place, it is also then that selection on behaviour and morphology is the strongest. Because *P. cristatus* and *P. montanus* cause *P. ater* to forage predominantly in the tree periphery during winter, interspecific competition maintains selection for a small body size of *P. ater* in sympatry on the mainland (Fig. 4).

### Alternative explanations

We think that the genetically determined increase in body size of *P. ater* on the islands is the result of adaptive evolution in response to the absence of competition from *P. cristatus* and *P. montanus*. But the large size may have come about for other reasons. Platt (1964) forcefully advocated the ‘method of multiple working hypotheses', placing focus on finding proof for exclusion. Here, we test 11 alternative hypotheses against available evidence. They are treated in turn below and can all be eliminated.

#### 1. Relict island populations reflecting historically widespread ancestral characteristics

*P. ater* is strictly bound to coniferous forest. But the four islands Bornholm, Sjælland, Anholt and Læsø were not planted with conifers until after 1850. In these recently founded populations, *P. ater* is equally large as conspecifics on Gotska Sandön and Gotland, both of which have had primeval coniferous forests since long-ago.

#### 2. Island *P. ater* derived from other populations than those that colonized the Swedish mainland

In view of the large geographical separation between the islands and their location to the east, south and west of the Swedish mainland, it is unlikely that the islands and the Swedish mainland got their populations from different sources. But most important, there is almost no variation in body size among continental *P. ater* over the northern Palearctic (Snow, 1954, 1955), so there is no nearby place from which large birds could have been recruited.

#### 3. Geographical trends or clinal variation in body size

*P. ater* has a continuous distribution across the northern Palearctic and shows great uniformity in body dimensions with little clinal variation (Snow, 1954, 1955). So there is no geographical trend that could explain the size difference between island and mainland tits.

#### 4. Founder effect

This refers to the reduction of genetic variation that occurs when a population is established by a small number of pioneers. When there are few individuals in a colonizing group, random processes increase the likelihood that the group brings with it a skewed gene sample that is not representative of the parent population. This may contribute to evolutionary divergence.

*P. ater* is sedentary the whole year round but performs irruptive migrations at irregular intervals. The islands were most likely colonized by mainland birds that took part in such irruptions. But the probability is only 0.5^8^=0.004 that all eight places, from which one or two congeners are absent, were colonized by larger than average-sized mainland birds by chance alone. So random founder events cannot account for the large size of *P. ater* in all eight places.

We will now look at the size of present-day migrants of *P. ater.* Judging by observations during an autumn irruption in 1990, migrants consist almost exclusively of juveniles. On that occasion, 716 *P. ater* were netted and ringed at Falsterbo Bird Observatory and all of them were first-year birds (Lennart Karlsson, personal communication).

The hand-wing and the tarsus of first-year birds on southbound autumn migration were measured by staff ornithologists at the Falsterbo Bird Observatory, who used the measuring technique described in the methods section herein. Hand-wing length of migrants was remarkably similar between samples in different years. The average length was 61.26 mm (s.d. 1.62) for 273 migrating *P. ater* measured in September and October 1982-1986 (Göran Walinder, personal communication) and 61.23 mm (s.d. 1.51) for 180 birds measured in September and October 1990 (Lennart Karlsson, personal communication). This gives an overall average of 61.25 mm for migrants. The hand-wing length of resident first-year birds that we measured on the mainland was 60.9 mm in SW Sweden, 61.0 mm in SE Sweden and 61.3 mm in SC Sweden, with an average of 61.0 mm for the pooled mainland sample (Table 2). Residents in SC Sweden thus had a longer hand-wing than migrants. But the hand-wing in the pooled mainland sample is 0.4% shorter than in migrants (61.0 vs 61.25 mm). By contrast, hand-wing length of island birds exceeds that of mainland residents by up to 4.3% (63.6 on Bornholm vs 61.0 mm on the mainland; Table 2).

The average length of the tarsus was 18.60 mm (s.d. 0.59) in the 180 autumn migrants measured in 1990 (Lennart Karlsson, personal communication). The tarsus length of resident first-year birds that we measured on the mainland was 18.4 mm in SW Sweden, 18.3 mm in SE Sweden and 18.2 mm in SC Sweden (Table 3), with an overall average of 18.34 mm for mainland birds. So, the tarsus was 1.4% longer in migrants than in mainland residents (18.60 vs 18.34 mm). This is a small difference as compared, for example, with the 6.3% that separate birds on the island of Gotland from mainland residents (19.5 mm vs 18.34; Table 3).

In conclusion, judging by the wing and tarsus measurements of present-day migrants, the islands were not colonized by big immigrants arriving from the mainland without further size change. The size of present-day migrants instead suggests that island colonizers were similar in size to mainland residents and that a size increase came about by local evolutionary adaptation following colonization.

#### 5. Genetic drift

This is divergence of allele frequencies and associated phenotype traits between separated populations through random processes. It is distinct from any divergence already present due to founder effects. Genetic drift arises from random survival and random allele sampling during reproduction and is more likely to result in accidental loss of alleles the smaller the population. It is distinct from divergence due to local adaptation by natural selection.

A random change of body size is equally likely in either direction. Therefore, the increased size of *P. ater* in all eight places studied has a probability of independent, random occurrence of 0.5^8^=0.004. In addition to the size changes in the predicted direction, also the magnitude of increase is in accord with predictions. Therefore, genetic drift can be ruled out as a cause for the consistent increase in body size.

#### 6. Inbreeding

Because populations of *P. ater* are smaller on the islands than on the mainland, there is a higher risk of inbreeding on the islands. Inbreeding will tend to increase homozygosity and the expression of recessive deleterious alleles, potentially leading to inbreeding depression and reduced vigour (Falconer, 1983). If body size were at all affected it would likely shift towards smaller rather than bigger – contrary to the case on the islands. A study of a wild population of another passerine bird, the collared flycatcher *Ficedula albicollis*, showed that inbreeding caused a significant reduction in fledgling skeletal size and in post-fledgling juvenile survival (Kruuk et al., 2002).

Another argument against inbreeding as a cause for the increased size of *P. ater* relates to differences in population size between islands. Gotland is the largest island, Anholt the smallest, and the area of coniferous forest on them is 1147 km^2^ and 3.0 km^2^, respectively (Table 6). If population size is taken to be proportional to the area of coniferous forest, there would be 400 times more individuals of *P. ater* on Gotland than on Anholt. The number is estimated to be about 80,000 on Gotland (SOF, 1990) so there would be about 200 on Anholt. Another census on Gotland estimated the population density of *P. ater* to 90–110 individuals per km^2^ of pine-dominated forest in January-February (Appendix in Alatalo et al., 1986). Estimates based on these numbers give similar results, namely 100,000–126,000 individuals on Gotland and 270–330 on Anholt. But although the population is 400 times larger on Gotland than on Anholt, *P. ater* is the same size on both islands.

**Table 6.**
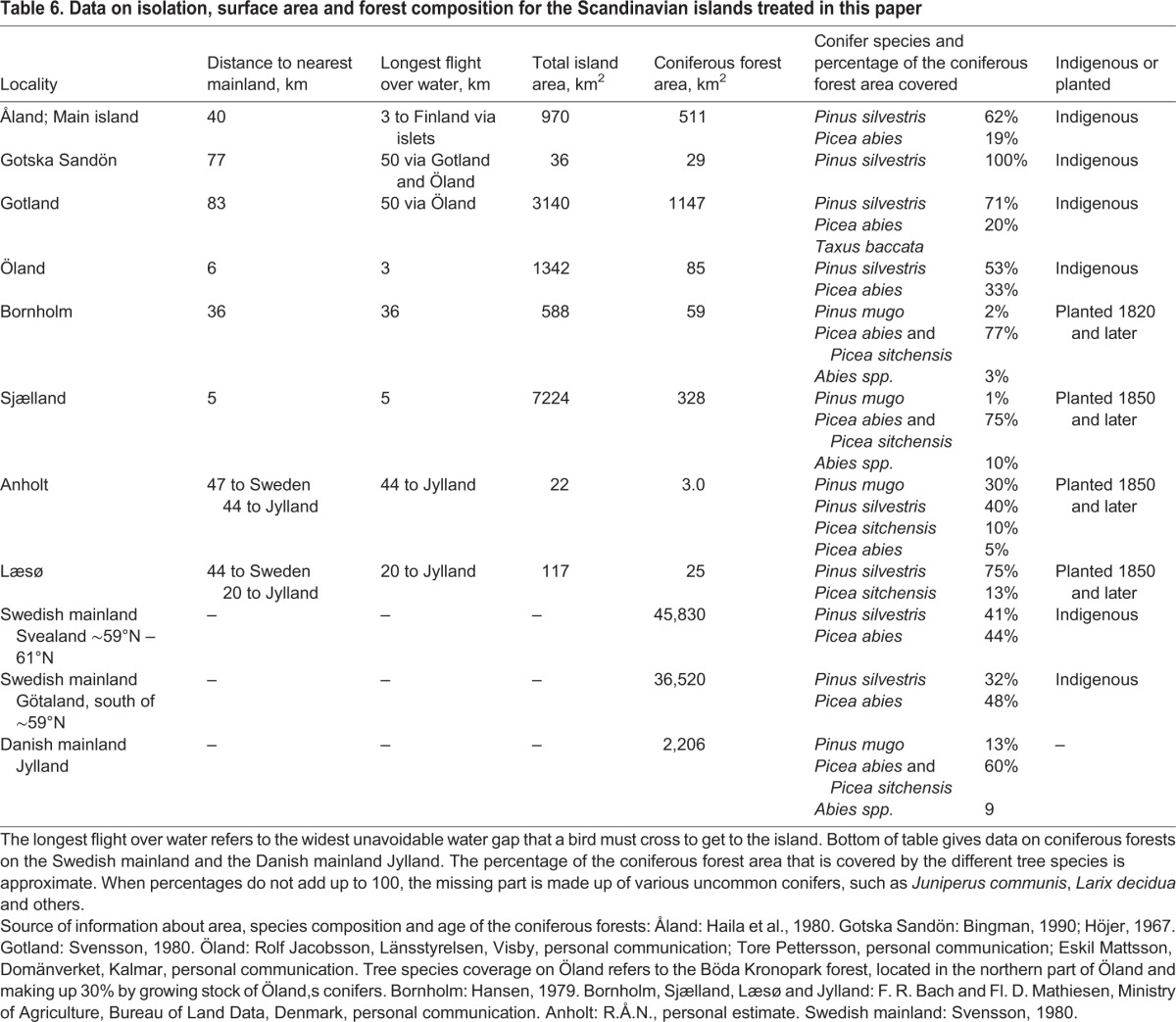
**Data on isolation, surface area and forest composition for the Scandinavian islands treated in this paper**

#### 7. Habitat

For *P. ater*, the absence of *P. cristatus* and *P. montanus* from the islands is the most important habitat difference from the Swedish mainland. Other than that, island habitats are often considered to be impoverished in relation to their mainland counterparts, implying that the abundance and diversity of food items are less on islands (Lack, 1976). If that were to affect body size, island birds might be smaller than mainland conspecifics due to direct effects on the phenotype during the nestling period, or because of evolutionary adaptation to poor food conditions. But *P. ater* is instead larger on the islands.

Island forests might have characteristics that favour a large body size in *P. ater*. But forest structure and tree species composition vary considerably between islands and there is no forest feature that is shared by all islands and distinguishes them from the mainland. As an example, the forest is 100% pine *Pinus silvestris* on Gotska Sandön, whereas spruce *Picea* spp. and *Abies* spp. make up 77% of the forests on Bornholm (Table 6). Yet *P. ater* is about the same size on both islands. The possibility that some feature of planted forests on the islands of Bornholm, Anholt and Læsø underlies the large body size is refuted by the fact that *P. ater* is as large also on the islands of Gotska Sandön and Gotland, the forests of which are indigenous and natural.

#### 8. Climate

In the thermoneutral zone of a warm-blooded animal there is no metabolic energy cost for thermoregulation. But below it, metabolic heat must be generated to maintain body temperature. For the same insulation and temperature gradient across the body surface, the rate of heat loss is proportional to body surface area. Among geometrically similar animals, area is proportional to volume^2/3^ so the rate of heat loss from an entire animal increases in proportion to mass^2/3^. But the rate of heat loss per unit body mass decreases with increasing body size as mass^2/3^/mass=mass^−1/3^. So, heat loss per unit body mass is larger the smaller the animal. Selection for reduced mass-specific metabolic costs for thermoregulation in temperate and cold regions therefore favours large body sizes. This is a likely explanation for the intraspecific trend of increasing body size among some warm-blooded vertebrates in colder parts of their distribution range – known as Bergmann's ecogeographical rule (Bergmann, 1847; Mayr, 1965). Another explanation is that the size trend arises because large animals have better fasting ability, which is particularly advantageous in cold and seasonal environments with periods of food scarcity (Murphy, 1985). Either way, selection for large size must be strongest in winter.

**Fig. 5.**
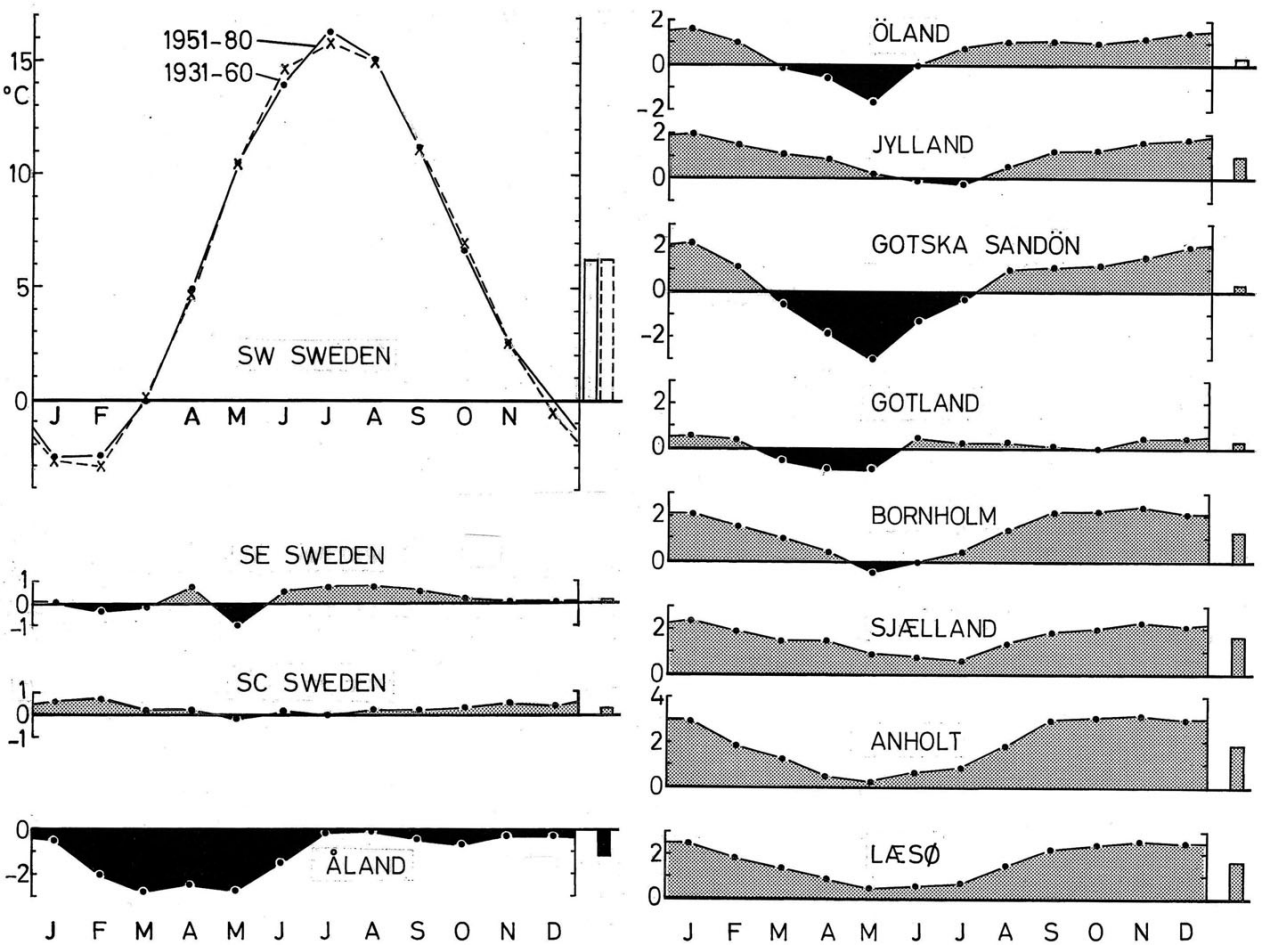
**Comparisons of mean monthly temperatures at the 12 places where we measured tits.** The diagram at top left shows the actual mean monthly temperature in degree centigrade at the reference locality in SW Sweden, averaged over each of two partly overlapping 30-year periods, 1931–1960 and 1951–1980. The other diagrams show local deviations from the mean monthly temperatures recorded in SW Sweden during the period 1931–1960. The vertical bars to the right of the top left diagram show the mean yearly temperature in SW Sweden for the two 30-year periods. Bars to the right of the other diagrams show local deviations from the mean yearly reference temperature in SW Sweden. Temperature was recorded at meteorological stations near the respective sampling place. Data were obtained from The Swedish Meteorological and Hydrological Institute, Norrköping, for the periods 1931–60 and 1951–80; The Danish Meteorological Institute, Copenhagen, for the period 1931–60; and The Finnish Meteorological Institute, Helsinki, regarding the island of Åland for the period 1951–80.

On the islands where *P. ater* occurs but *P. cristatus* and *P. montanus* are absent, the winter climate is considerably milder than on the Swedish mainland, except that Gotland is like the mainland (Fig. 5). If reduction of mass-specific energy costs for thermoregulation in winter were important, mainland birds would be larger than their island conspecifics. But body mass and all linear measurements of *P. ater* are smaller on the Swedish mainland than on the islands where *P. cristatus* and *P. montanus* are lacking (Figs 1 and 3). And on the island of Åland, where all three species coexist, *P. ater* is the same small size as on the Swedish mainland even though Åland is the coldest among the islands (except for Gotska Sandön in May) and colder in all seasons than the Swedish mainland (Figs 1, 3 and 5). So, climate cannot explain the large size of *P. ater* on the islands.

#### 9. Snow cover

A field study in pine forest *Pinus* spp. in the eastern Pyrenees showed that *P. ater* foraged mainly in the canopy periphery under snow-free conditions (Brotons, 1997), just like it does on the Swedish mainland. But when there is snow, the distal branch parts became less accessible. It then shifted to the inner parts of trees, using trunks and thick branches as the main foraging space. This change of foraging sites was associated with a shift to less energy-demanding locomotion modes (Brotons, 1997).

Small body size furthers manoeuvrability and cuts costs of the energy-expensive locomotion modes that are required in the canopy periphery. But when snow forces *P. ater* to forage on the inner tree parts, where it can use easy locomotion modes, selection for a small body size is relaxed. During the snowy period, the balance between conflicting selection pressures therefore shifts temporarily towards a larger body size (Fig. 4).

The Swedish mainland and the islands Åland, Gotska Sandön and Gotland have longer snow periods than Bornholm, Sjælland, Anholt and Læsø (Table 7). A large body size should therefore be more favoured in the former places. But *P. ater* is small on the Swedish mainland and the island of Åland, where also *P. cristatus* and *P. montanus* occur, and large on the other islands, all of which lack *P. cristatus* and *P. montanus*. This is contrary to expectations based on the length of the snowy season so differences in snow cover cannot explain the large size of *P. ater* on the islands.

**Table 7.**
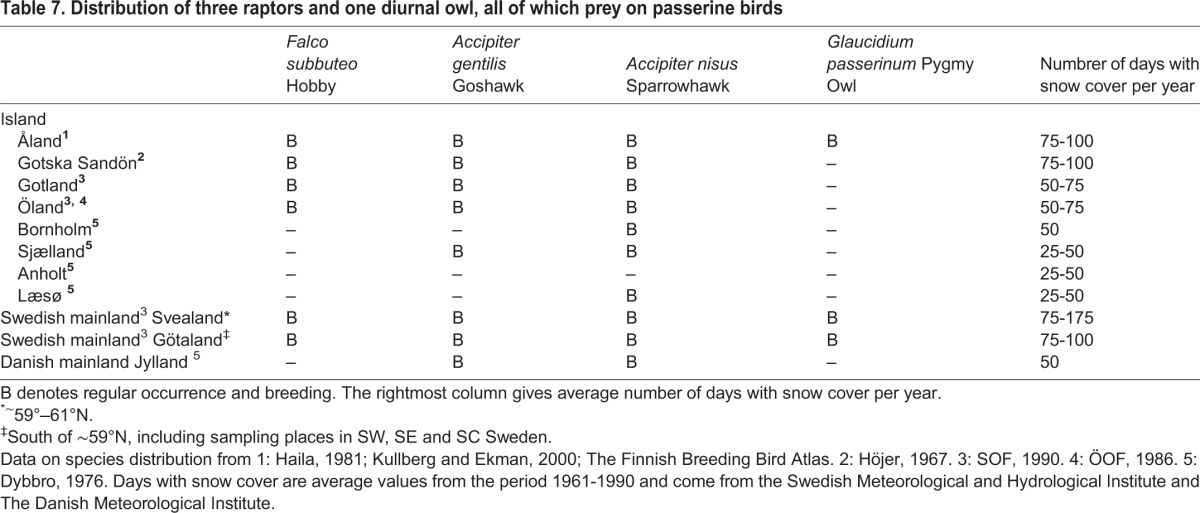
**Distribution of three raptors and one diurnal owl, all of which prey on passerine birds**

#### 10. Nest holes

If nest holes are in short supply, large individuals would get a competitive advantage. At a field site in Belgium, the mean size of *Parus major* showed a decreasing trend in the period 1962–1975. This was concomitant with the provision of an abundance of nest boxes. Therefore, the progressive reduction in body size during this period was interpreted to be the result of relaxed competition for nest holes, whereby big individuals lost some of their advantage (Dhondt et al., 1979). But apart from using tree holes, *P. ater* nests readily in holes under tree roots and stones, so it is unlikely that there is a shortage of nest holes either on the mainland or on the islands.

#### 11. Predation

When tits forage in the canopy periphery, they are exposed to high predation risk, whereas foraging closer to the trunk gives structural protection from raptors and day-hunting owls (Ekman, 1986; Kullberg, 1995; Götmark and Post, 1996). Therefore, if predation pressure differs between islands and the mainland, island tits might change their use of foraging zones in trees. And that entails a shift in locomotion mode, which in turn may alter selection on body size.

Here follows a review of predation on coniferous forest tits and how predators influence their foraging behaviour and thence the selection on morphology. We focus on raptors and a day-hunting owl whose hunting success depends on where in trees their prey is. Night-hunting owls and nest predators are thus left out.

A study in SW Sweden found that the sparrowhawk *Accipiter nisus* takes few coniferous forest tits in winter but preys more on them in summer (Table 2 in Ekman, 1984). Another study, performed in summer in SW Sweden, compared the proportion of different bird species in the diet of *A. nisus* with their relative abundance in the local bird community. The hawk took somewhat fewer *P. montanus* than expected whereas the proportion of *P. ater* and *P. cristatus* in its diet was much lower than expected from their relative abundance on the hawks' hunting grounds (Götmark and Post, 1996). *P. montanus* and *P. cristatus* forage in relatively predator-safe tree parts, which may explain their low frequency in the hawk diet. But the still lower predation rate on *P. ater* runs counter to expectations based on its use of exposed foraging sites.

The pygmy owl *Glaucidium passerinum* hunts by day and is responsible for a considerable proportion of the winter mortality of *P. cristatus* and *P. montanus* in SW Sweden (Ekman et al., 1981; Ekman, 1984, 1986). In a study area where *P. cristatus* and *P. montanus* were individually colour-banded, the tits were very sedentary in winter and did not leave the territory of their respective flock. Any disappearance from the winter territory in the period November-March was therefore attributed to mortality. Among those that disappeared, 19% of the number of *P. cristatus* and 24% of *P. montanus* were found – and identified by their rings – in regurgitated pellets in winter roosting holes of *G. passerinum* (Table 5 in Jansson et al., 1981). These predation rates are probably underestimates because the remains of some prey must have been missed.

When *G. passerinum* hunts birds, it strikes from above, launching the attack from perches that are higher up than the focal prey (Kullberg, 1995). Therefore, predation risk is greater in the low and outer parts of a tree than higher up or in cover from branches and twigs in the inner tree zones (Ekman, 1986; Kullberg, 1995).

In a study in SW Sweden, the proportion of various bird species in the diet of *G. passerinum* was compared with their relative abundance in the local habitat. *P. montanus*, which forages near the trunk, was preyed upon about as expected from its relative abundance, whereas *P. cristatus*, which forages farther out, was preyed upon more than expected and *P. ater* even more so, which is in accord with its use of exposed foraging sites (Table 2 in Ekman, 1986). A survey of six field studies in Norway, Sweden, Finland and Russia likewise showed that *G. passerinum* preys consistently more heavily on *P. ater* and *R. regulus* and less on *P. cristatus* and *P. montanus* than expected from their relative abundance in the local bird community (Suhonen et al., 1993). Empirical results thus confirm that *G. passerinum* takes disproportionately many *P. ater* – as expected in view of its exposed foraging sites – whereas *A. nisus* unexpectedly takes disproportionately few *P. ater* (see above; Götmark and Post, 1996).

Predation by *G. passerinum* has been shown to determine the choice of foraging sites by tits to various extents depending on the availability of small rodents, which are preferred prey of the owl. In one winter when rodent populations were at a peak level in Finland, voles and shrews *Sorex* spp. together made up 98% and birds 2% of the diet of *G. passerinum*. In the next winter, the rodent populations had crashed and rodent trap-catches dropped to 0.6% of the value in the previous winter, from 32 to 0.2 captured individuals per 100 trap-nights. At this rodent population low, *G. passerinum* switched to eating birds, after which 40% of its winter diet consisted of rodents and shrews while birds made up 60%. Following this huge shift in the owl's diet, *P. cristatus* and *P. montanus* responded behaviourally and foraged less on the outer, exposed regions of spruce trees and more often on the inner parts of branches (Suhonen, 1993).

*P. cristatus* and *P. montanus* are claimed to be socially dominant over *P. ater* and to exclude it from their foraging zones, which leaves it to forage in the risky outer canopy (Hogstad, 1978; Alatalo and Moreno, 1987; Suhonen et al., 1993; Suhonen, 1993). Rank order has been observed to determine feeding site partitioning also at the intraspecific level. In *P. montanus*, dominant adults expel subordinate first-year birds from the upper tree regions in winter, leaving them to forage lower in trees where they are more vulnerable to predation (Ekman and Askenmo, 1984).

Table 7 shows the distribution of potential predators on *P. ater*. The hobby *Falco subbuteo* is specialised on open-air hunting and the goshawk *Accipiter gentilis* mostly takes large prey, so both have little impact on tits. *A. nisus* is a breeding resident on the mainland of Denmark, Norway and Sweden and on all islands except Anholt. *G. passerinum* is a breeding resident on the mainland of Norway, Sweden and Finland. It has also been recorded on the island of Åland (Haila, 1981; Kullberg and Ekman, 2000), but the population density is low there and its breeding status uncertain (The Finnish Breeding Bird Atlas). It is absent from all the other islands as well as from the Danish mainland Jylland.

The islands have different numbers of resident raptor species (Table 7). There are three on Gotska Sandön and Gotland, two on Sjælland, one on Bornholm and Læsø and none on Anholt. And yet, *P. ater* has increased in size to a similar extent on all six islands (Figs 1 and 3; Tables 2-4). So, the large size of *P. ater* on the islands is not driven by predation. But its body size on the mainland might be influenced indirectly by predators in so far as they affect niche partitioning among tits to the effect that *P. cristatus* and *P. montanus* forage in the safer inner tree regions. By force of their putative dominance, they may expel *P. ater* from the inner parts, leaving it to forage in the exposed tree periphery (Hogstad, 1978; Alatalo and Moreno, 1987; Suhonen et al., 1993; Suhonen, 1993). Once there, it is subject to selection for agility and a small body size.

## CONCLUSION

None of the alternative explanations is supported, so our *a priori* hypothesis is upheld. Because the large size of *P. ater* on the islands is genetically determined, we conclude that it is the result of evolutionary adaptation, due ultimately to the local absence of the larger competitors *P. cristatus* and *P. montanus.* Judging by the wide geographical separation of the islands, the large size cannot be the result of a single, local, evolutionary event, followed by dispersal to the other islands. Instead, the size increase must have evolved independently on separate islands after colonization.

The enlarged body size has probably evolved because when *P. cristatus* and *P. montanus* are absent, *P. ater* takes over the vacant niche space in the inner tree parts. The takeover is promoted (1) by food in the vacant space (2) and by the absence of competition with the ordinary niche holders. The extended niche space enables *P. ater* to reach high population densities on the islands. Because the food base is widened, competition for food may not increase, but intraspecific interference competition is likely to increase, which strengthens selection for a large body size (Fig. 4). And as indicated by the large size of *P. cristatus* and *P. montanus*, which mostly forage on the thick parts of branches, a large body is better adapted to places where food can be obtained by pecking and tearing in bark and wood (Fig. 4).

In sympatry with *P. cristatus* and *P. montanus* on the Swedish mainland, *P. ater* forages predominantly in the outer tree parts. Foraging on the fine and pliant distal twigs in the tree periphery requires frequent hanging underneath branches and many short flights and hovering bouts. For mechanical and aerodynamic reasons a small body size improves agility and manoeuvrability and reduces energy cost of locomotion. Selection therefore favours a small size in *P. ater* on the mainland.

But when *P. ater* exploits the vacant foraging sites of *P. cristatus* and *P. montanus* on the islands, it also adopts their easier foraging techniques and locomotion modes, which reduces demands for manoeuvrability and power output. In addition, after widening its foraging zone in trees, *P. ater* cuts down on its exploitation rate of the canopy periphery, where high manoeuvrability and energy-expensive locomotion modes are required. Selection for a small body size is therefore reduced (Fig. 4).

On the six islands where *P. ater* occurs alone, it is near the average size as calculated across *P. ater*, *P. cristatus* and *P. montanus* in sympatry on the Swedish mainland (Figs 1-3). So, in the absence of the two larger competitors, *P. ater* has evolved in the direction of the absentees on the six islands and become a medium-sized generalist.

In two other places, *P. ater* coexists with one coniferous forest congener – with *P. montanus* on the island of Öland and with *P. cristatus* on the Danish peninsula Jylland. *P. ater* is larger in both places than on the Swedish mainland. But the size increase is less on Öland and Jylland than on the six islands from which *P. cristatus* as well as *P. montanus* are absent.

The body size of *P. ater* is enlarged in all eight places where it was expected. So here, evolution of local adaptation could be predicted both regarding direction (qualitatively) and the amount of change (quantitatively). And it has taken place independently and in parallel in geographically separated places (repeatability). The large size has evolved within the last 100 years on the four islands Bornholm, Sjælland, Anholt and Læsø because the coniferous forest habitat required by *P. ater* was totally lacking before then.

A corollary of the niche extension and evolutionary size increase in *P. ater* when it is geographically isolated from *P. cristatus* and *P. montanus*, is that when all three coexist, *P. cristatus* and *P. montanus* obviously exclude *P. ater* from the inner tree zones, either by virtue of being larger and socially dominant or by reducing food to levels at which it is not profitable for *P. ater* to forage. It leaves *P. ater* to exploit the tree periphery, which requires high manoeuvrability and agility. This keeps up selection for a small body size of *P. ater* on the mainland (Fig. 4).

Predators may play an indirect role in maintaining the small size of mainland *P. ater* in so far as they influence niche partitioning among tits to the effect that *P. cristatus* and *P. montanus* forage in the safer inner tree parts from where they drive *P. ater* into the tree periphery where small size is selected for.

## MATERIALS AND METHODS

We measured birds at the 12 places shown in Fig. 1. Three are located on the Swedish mainland, one on the Danish mainland Jylland and eight on islands. The islands are the Finnish island of Åland, the three Swedish islands Gotska Sandön, Gotland and Öland and the four Danish islands Bornholm, Sjælland, Anholt and Læsø (Fig. 1). The sampling places in SW, SE and SC Sweden are located in a large, continuous, coniferous forest region in which *P. ater*, *P. cristatus* and *P. montanus* coexist. The mainland locations were chosen so as to match as far as possible the geographic separation between the investigated islands (Fig. 1). The geographic distribution of *P. ater* reaches north to about latitude 63°N in Sweden and to about 62°N in Norway and Finland. The Finnish island of Åland, located at 60°N in the Baltic Sea, is our northernmost sampling locality.

We carried out the fieldwork in four consecutive years, from 1979 to 1982. It was performed between August 9 and October 8 in all years and in all places except for the island of Gotska Sandön, where we were during the breeding period in June, measuring adult birds only. We used song playback and mist-nets to capture birds. They were measured immediately at the capture site and were then set free. At each locality, we shifted net positions repeatedly in order to sample from different flocks. Between 8 and 37 living specimens of *P. ater* were measured at each of the 12 sampling localities shown in Fig. 1. We also measured a total of 11 *P. cristatus* and 14 *P. montanus* at the SW and SE Swedish sampling places.

‘First-year birds’ refers to full-grown birds born in the breeding season of the same calendar year. The tibiotarsus and tarsometatarsus are fully grown in *P. ater* by day 14 after hatching (own observation), whereas flight feathers and the bill grow for some time still. The young fledge in late June so all growth has ceased long before our fieldwork started in August.

First-year birds greatly outnumber adults in our autumn samples. Therefore, and because wing and tail feathers do not reach adult size until the summer moult in the second calendar year (Svensson, 1992), we use only first-year birds in all comparisons except where otherwise stated. The Gotska Sandön sample only contains adults, which we compare with adults from the mainland. First-year birds are easy to tell from adults by plumage (Svensson, 1992). But there is no external character that permits reliable sexing, so the sexes are pooled in each age category. The handling of birds in this study complies with all relevant institutional and national animal welfare laws, guidelines and policies.

### Measurements taken

We weighed birds with a 30 g Pesola spring balance, read to the nearest 0.1 g, and used rulers to measure wing span to the nearest 1 mm and hand wing and tail to the nearest 0.5 mm. The other linear measurements were taken with a plastic dial calliper rule, read to the nearest 0.1 mm. Wing area was measured from photographs taken of birds with outstretched wings as described below. We projected an enlarged image onto paper, draw the wing outline and measured wing area with a polar compensating planimeter. We made three planimeter tracings of the same drawing of each individual and calculated the average area.

We collaborated on all fieldwork. But to eliminate variation due to possible differences in measuring technique, one of us made all measurements in the field whereas the other measured all wing areas from photographs. Whenever uncertainty arose how to round off a reading, we counteracted unintentional bias by consistently rounding in the opposite direction to that expected from our working hypothesis.

*Body mass*: We weighed most birds within one hour of capture. But in a few cases, weighing was delayed for up to two hours due to the handling of a large catch.

*Wing area*: Wing area is the area of both wings and the portion of body in between as projected onto the wing-chord plane. In flight, the reduced pressure over the wings is retained over the body portion between the wings so it is part of the lift-producing area. We measured wing areas from photographs taken in dorsal view, perpendicular to the wing chord plane, of birds held against a board with a scale. We held the wings by the wrist and extended them moderately as they are judged to be in mid-downstroke. We also rotated the handwings forward to straighten and align the leading edges of the wings and spread the flight feathers evenly but without disrupting their natural backward and ventral curvature.

*(Wing area)^0.5^*: Among geometrically similar animals, areas increase as a representative length squared, so areas in different sized animals cannot be directly compared with linear measurements. Therefore, we calculated the square root of the wing area to obtain another linear measure of wing size – in effect, the length of the side of a quadrate with the same area as the wings. The square root of wing area is not listed in any table and is used only to calculate a compound measure of wing linear size for the diagrams in Fig. 1.

*Wing span*: The bird was placed on its back and the wings were stretched moderately as described under ‘wing area’. Distance was measured from wingtip to wingtip without disrupting the natural ventral and backward curvature of the feathers that form the wingtip.

*Hand-wing length*: Distance from the wing-bend at the wrist to the wingtip. The wing was folded and the ruler was applied to the wing's ventral side with the bent wrist pressed against the ruler's zero-stop. The maximum hand-wing length was obtained by pressing the primary feathers flat against the ruler to eliminate their ventral curvature and by gently pushing backwards on the wing's leading edge near the alula and moving the feather tips forward to eliminate the lateral curvature.

*Wing loading*: We calculated wing loading as body weight in Newton divided by wing area in m^2^.

*Wing aspect ratio*: It is the ratio *B*/*c* between wingspan *B* and the mean wing chord *c*, and was calculated as wing span squared divided by wing area *S*; *B*^2^/*S*=*B*^2^/(*B*×*c*)=*B*/*c*.

*Tail length*: Distance from root to tip of the tail while it is folded and straightened along the ruler. The ruler's bevelled edge was placed between the tail feathers and the under tail-coverts and pushed gently against the roots of the two central tail feathers.

*Tibiotarsus length*: Distance from the top of the knee to the ventral side of the tarsometatarsus at the joint between tibiotarsus and tarsometatarsus when the knee and the tibiotarsus-tarsometatarsus joints are set at 90°. Care was taken to align the measurement with the long axis of the tibiotarsus.

*Tarsus (=tarsometatarsus) ‘short’ length*: Distance from the notch underneath the tibiotarsus-tarsometatarsus joint, when it is bent 90°, to the outer edge of the last complete scale on the dorsal side of tarsomtetatarsus, just before the toes.

*Tarsus (=tarsometatarsus) ‘long’ length*: Distance from the posterior surface of the tibiotarsus at the tibiotarsus-tarsometatarsus joint, when it is bent 90°, to the dorsal side of the 3rd toe when it is bent 90° backwards relative to the tarsometatarsus. In *P. ater*, this distal measuring point coincides with the outer edge of the last complete scale on the dorsal side of the tarsometatarsus, used in the previous measurement. Care was taken to align the measurement with the long axis of the tarsometatarsus.

*Foot length*: Distance, measured on the dorsal side of the foot, from the edge of the skin at the base of the claw of the middle forward toe (#3) to the edge of the skin at the base of the claw of the hind toe (#1) when the toes are gently stretched forwards and backwards, respectively. The claws are not included in the measurement since they may be worn down to various extents.

*Bill length*: Length from the bill tip to the frontonasal angle at which the calliper jaw (for measuring inner dimensions) stops when it is gently slid backwards along the bill's culmen. We took a projected measurement parallel to the bill's cutting edge because if one measures obliquely, directly from the bill tip, it is difficult to locate the flat frontonasal angle that is concealed by feathers.

*Bill width*: Width measured at the middle of the nostrils.

*Bill depth*: Bill height at the middle of the nostrils measured at 90° to the bill's cutting edge. Care was taken not to press the bill beyond its natural closure.
